# Supplementary figures and images for: Transformative optimisation of agricultural land use to meet future food demands
Source: PeerJ. 2013 Oct 24;1:e188. doi: 10.7717/peerj.188 (PMC3817586; doi:10.7717/peerj.188)

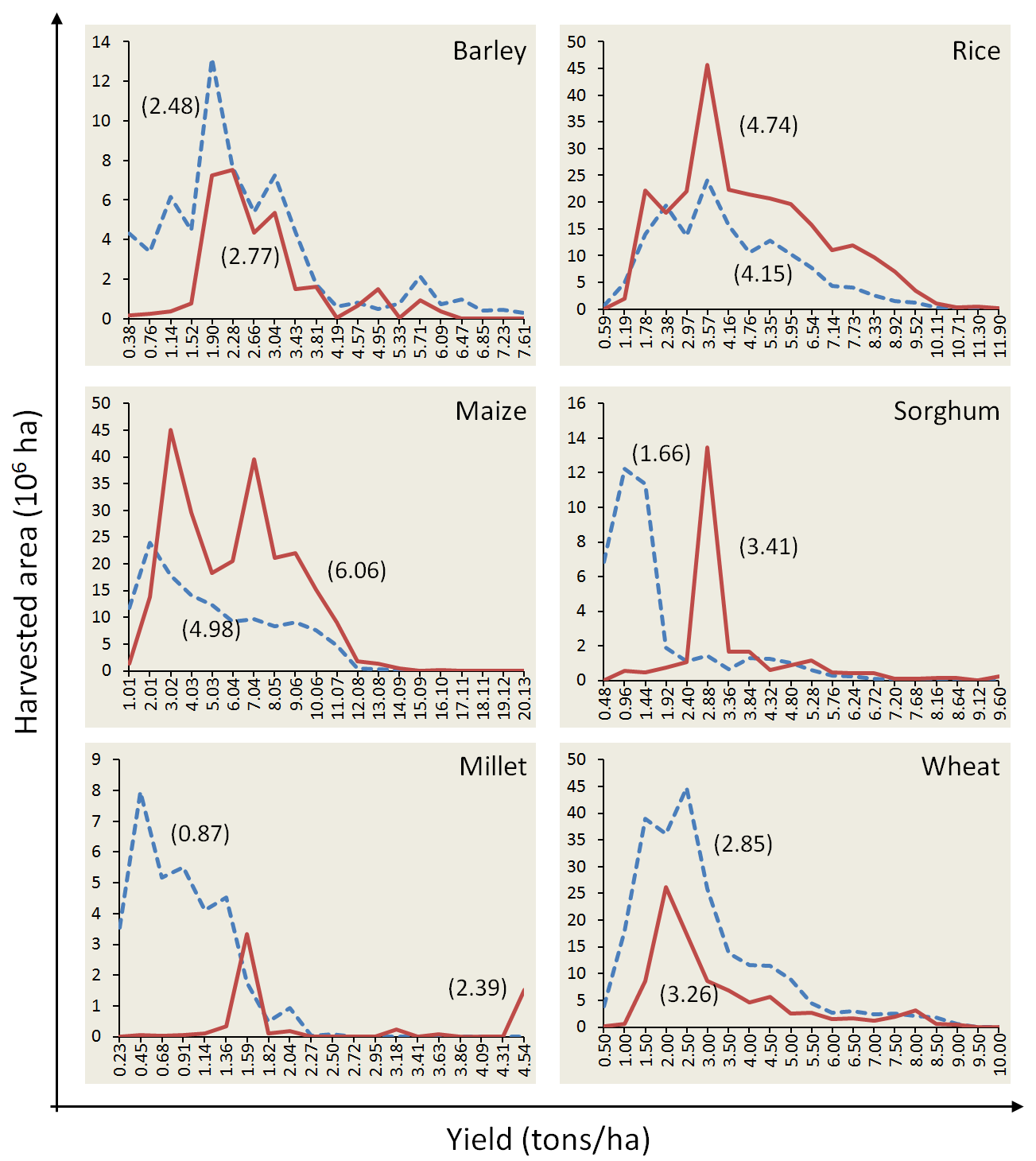

Supplement: Figure S1 — Dashed blue lines indicate current land use scenario; red lines indicate optimal land use scenario; numbers on individual x-axes indicate annual yield; numbers in parentheses indicate area-weighted average annual yield. [file peerj-01-188-s001.png]

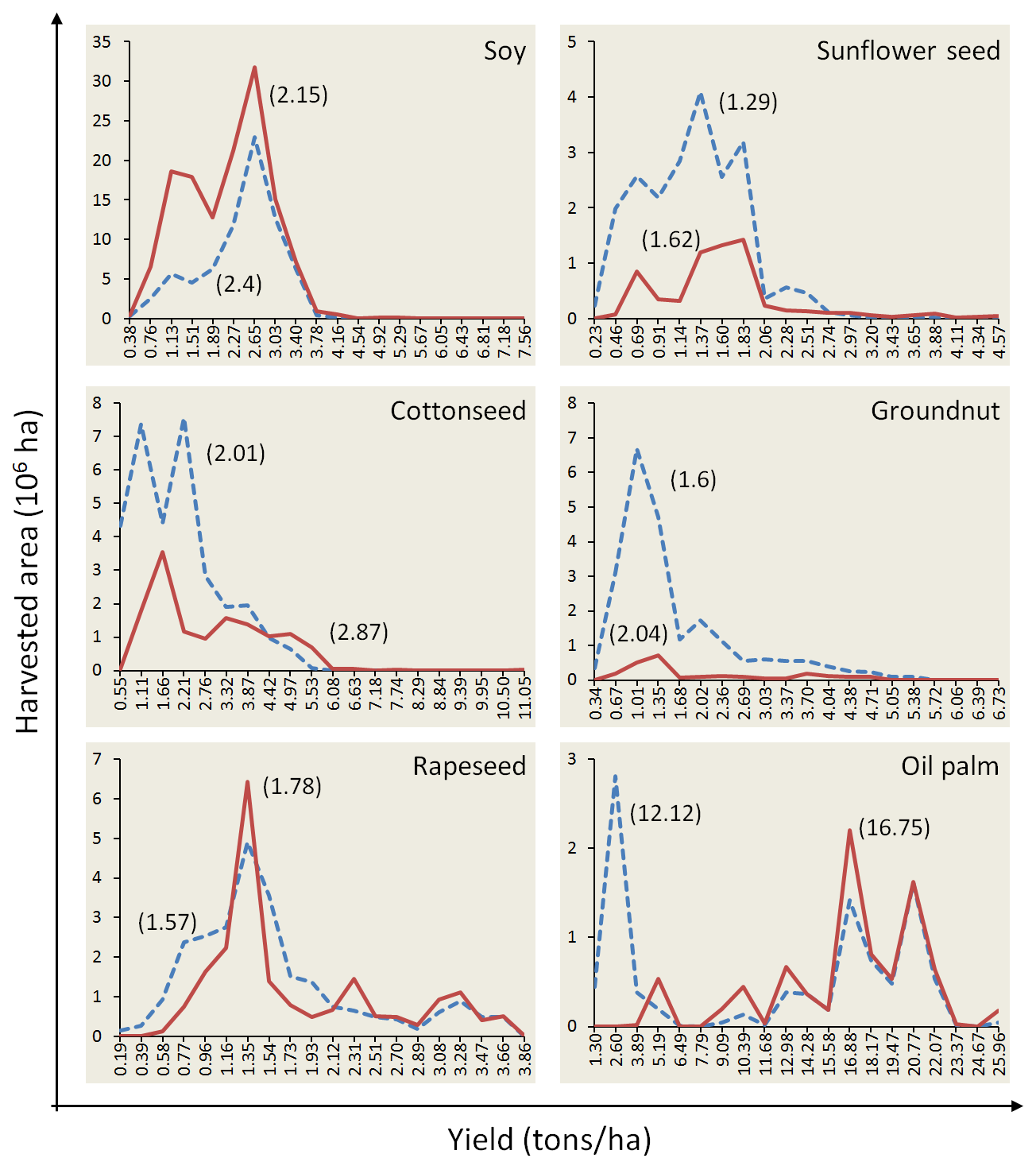

Supplement: Figure S2 — Dashed blue lines indicate current land use scenario; red lines indicate optimal land use scenario; numbers on individual x-axes indicate annual yield; numbers in parentheses indicate area-weighted average annual yield. [file peerj-01-188-s002.png]
