# Supplementary material for: Transformative optimisation of agricultural land use to meet future food demands
Source: PeerJ. 2013 Oct 24;1:e188. doi: 10.7717/peerj.188 (PMC3817586; doi:10.7717/peerj.188)
Supplement: Table S1 [file peerj-01-188-s003.docx]

**Table S1. Harvested area of cereal crops in 171 countries under current and optimal land-use allocation options (all values in 10^6^ ha).**

|  |  | Barley | |  | Maize | |  | Millet | |  | Rice | |  | Sorghum | |  | Wheat | |
| --- | --- | --- | --- | --- | --- | --- | --- | --- | --- | --- | --- | --- | --- | --- | --- | --- | --- | --- |
| Country |  | Current | Optimal |  | Current | Optimal |  | Current | Optimal |  | Current | Optimal |  | Current | Optimal |  | Current | Optimal |
| China |  | 1.19 | 0.3 |  | 22.83 | 8.3 |  | 1.13 | 0 |  | 28.64 | 65.99 |  | 0.95 | 0.93 |  | 25.14 | 4.36 |
| United States |  | 1.99 | 2.03 |  | 28.93 | 53.36 |  | 0.17 | 0 |  | 1.41 | 0.39 |  | 3.16 | 0.71 |  | 22.08 | 1.24 |
| India |  | 0.75 | 8.54 |  | 6.01 | 12.3 |  | 12.66 | 0.06 |  | 43.96 | 54.52 |  | 9.77 | 0.22 |  | 27.19 | 24.71 |
| Russia |  | 10.57 | 0.37 |  | 0.88 | 24.55 |  | 0.81 | 0.02 |  | 0.24 | 5.37 |  | 0 | 0.47 |  | 22.06 | 3.8 |
| France |  | 1.62 | 0 |  | 1.63 | 8.46 |  | 0 | 0 |  | 0.03 | 0 |  | 0.08 | 0 |  | 5.16 | 0.06 |
| Indonesia |  | 0 | 0 |  | 2.9 | 0.19 |  | 0 | 0 |  | 11.28 | 13.99 |  | 0 | 0 |  | 0 | 0 |
| Australia |  | 5.05 | 7.91 |  | 0.16 | 10.39 |  | 0.08 | 0.11 |  | 0.31 | 5.25 |  | 1.12 | 0.29 |  | 17.8 | 0.58 |
| Canada |  | 4.51 | 1.63 |  | 1.23 | 15.28 |  | 0 | 0 |  | 0 | 0 |  | 0 | 0 |  | 11.27 | 0.09 |
| Brazil |  | 0.15 | 0.06 |  | 9.3 | 8.02 |  | 0 | 0 |  | 2.72 | 4.6 |  | 0.44 | 1.01 |  | 1.47 | 0.38 |
| Bangladesh |  | 0.01 | 0 |  | 0.02 | 0.61 |  | 0.06 | 0 |  | 11.02 | 11.1 |  | 0 | 0 |  | 0.83 | 0.23 |
| Argentina |  | 0.23 | 0 |  | 2.73 | 8.58 |  | 0.05 | 0 |  | 0.19 | 0.67 |  | 0.62 | 0.32 |  | 5.76 | 0 |
| Pakistan |  | 0.14 | 0.01 |  | 1.04 | 0.03 |  | 0.49 | 0 |  | 2.51 | 4.59 |  | 0.41 | 0 |  | 8.84 | 8.8 |
| Germany |  | 1.71 | 0.01 |  | 0.3 | 3.14 |  | 0 | 0 |  | 0 | 0 |  | 0 | 0 |  | 2.4 | 1.26 |
| Vietnam |  | 0 | 0.01 |  | 0.53 | 1.23 |  | 0 | 0 |  | 6.34 | 5.64 |  | 0 | 0 |  | 0.01 | 0 |
| Egypt |  | 0.07 | 0.03 |  | 1.49 | 1.24 |  | 0 | 0 |  | 0.79 | 2.46 |  | 0.36 | 0.25 |  | 1.49 | 0.22 |
| Turkey |  | 3.33 | 1.49 |  | 0.55 | 6.46 |  | 0.01 | 0.29 |  | 0.06 | 4.56 |  | 0 | 0 |  | 8.96 | 0.12 |
| Thailand |  | 0 | 0.52 |  | 1 | 8.58 |  | 0.01 | 0 |  | 9.67 | 1.68 |  | 0.09 | 0 |  | 0 | 0 |
| Ukraine |  | 4.2 | 0.01 |  | 1.36 | 2.95 |  | 0.27 | 0 |  | 0.02 | 7.88 |  | 0 | 0 |  | 5.97 | 0.98 |
| Mexico |  | 0.22 | 0.66 |  | 6.21 | 1.51 |  | 0 | 0 |  | 0.07 | 2.07 |  | 1.95 | 3.78 |  | 0.56 | 0.99 |
| Iran |  | 2.09 | 0.11 |  | 0.27 | 10.32 |  | 0.05 | 0.03 |  | 0.71 | 0.36 |  | 0 | 0 |  | 7.9 | 0.2 |
| Others (below) |  | 27.65 | 9.03 |  | 40.35 | 53.88 |  | 18.41 | 5.63 |  | 27.89 | 63.26 |  | 21.44 | 16.26 |  | 58.6 | 46.29 |
| United Kingdom |  | 1.32 | 0.01 |  | 0 | 0 |  | 0 | 0 |  | 0 | 0 |  | 0 | 0 |  | 1.8 | 3.12 |
| Myanmar |  | 0 | 0.01 |  | 0.24 | 0.07 |  | 0.24 | 0 |  | 5.94 | 6.44 |  | 0 | 0 |  | 0.11 | 0.01 |
| Spain |  | 3.31 | 0 |  | 0.47 | 5.98 |  | 0 | 0 |  | 0.13 | 0.07 |  | 0 | 0.11 |  | 2.27 | 0.02 |
| Italy |  | 0.27 | 0 |  | 1.2 | 3.22 |  | 0 | 0 |  | 0.23 | 0.02 |  | 0.04 | 0.17 |  | 1.67 | 0 |
| Nigeria |  | 0 | 0 |  | 3.8 | 1.12 |  | 4.8 | 0.08 |  | 2.24 | 2.81 |  | 5.85 | 0.07 |  | 0.04 | 12.67 |
| Romania |  | 0.45 | 0.35 |  | 2.91 | 4.65 |  | 0.01 | 0 |  | 0 | 0.1 |  | 0 | 0.01 |  | 2.03 | 0.29 |
| Philippines |  | 0 | 0 |  | 2.47 | 0.37 |  | 0 | 0 |  | 3.66 | 5.75 |  | 0 | 0 |  | 0 | 0 |
| Kazakhstan |  | 1.86 | 0.16 |  | 0.12 | 1.14 |  | 0.12 | 0 |  | 0.09 | 0.08 |  | 0 | 11.7 |  | 10.98 | 0.08 |
| Poland |  | 1.15 | 0.01 |  | 0.16 | 3.93 |  | 0 | 0 |  | 0 | 0 |  | 0 | 0 |  | 2.64 | 0.01 |
| Hungary |  | 0.35 | 0 |  | 1.18 | 2.61 |  | 0.01 | 0 |  | 0 | 0 |  | 0.01 | 0 |  | 1.09 | 0.03 |
| Syria |  | 3.39 | 0.06 |  | 0.13 | 4.9 |  | 0 | 0.06 |  | 0 | 0.49 |  | 0.01 | 0.05 |  | 4.28 | 2.26 |
| Japan |  | 0.07 | 0.01 |  | 0 | 0 |  | 0 | 0 |  | 1.5 | 1.72 |  | 0 | 0 |  | 0.17 | 0 |
| South Africa |  | 0.13 | 0.02 |  | 3.04 | 1.41 |  | 0.04 | 0 |  | 0 | 0.45 |  | 0.13 | 1.06 |  | 0.83 | 1.24 |
| Saudi Arabia |  | 0.19 | 1.95 |  | 0.02 | 0.1 |  | 0.03 | 0.11 |  | 0 | 0 |  | 0.39 | 0.09 |  | 2.17 | 0.54 |
| Denmark |  | 0.82 | 0.02 |  | 0 | 0.17 |  | 0 | 0 |  | 0 | 0 |  | 0 | 0 |  | 0.69 | 1.31 |
| Morocco |  | 3.74 | 0.18 |  | 0.47 | 1.71 |  | 0.02 | 3.23 |  | 0.01 | 2.62 |  | 0.03 | 0 |  | 4.26 | 0.78 |
| Serbia |  | 0.13 | 0 |  | 1.17 | 0.62 |  | 0 | 1.25 |  | 0 | 0.02 |  | 0 | 0.04 |  | 0.64 | 0.02 |
| South Korea |  | 0.07 | 0 |  | 0.02 | 0 |  | 0 | 0 |  | 0.95 | 1.05 |  | 0 | 0 |  | 0.01 | 0 |
| Uzbekistan |  | 0.1 | 0 |  | 0.05 | 2.13 |  | 0 | 0 |  | 0.13 | 0.03 |  | 0.01 | 0.02 |  | 1.98 | 0.09 |
| Nepal |  | 0.02 | 0.05 |  | 0.66 | 0.03 |  | 0.2 | 0 |  | 1.35 | 2.7 |  | 0 | 0 |  | 0.66 | 0.12 |
| Bulgaria |  | 0.31 | 0.51 |  | 0.47 | 0.87 |  | 0.01 | 0 |  | 0.01 | 0.39 |  | 0.01 | 0 |  | 1.19 | 0.23 |
| Sweden |  | 0.76 | 0.05 |  | 0 | 0 |  | 0 | 0 |  | 0 | 0 |  | 0 | 0 |  | 0.51 | 1.23 |
| Czech Republic |  | 0.41 | 0 |  | 0.05 | 1.2 |  | 0 | 0 |  | 0 | 0 |  | 0 | 0 |  | 0.75 | 0.02 |
| Ethiopia |  | 0.64 | 0.12 |  | 1.2 | 2.23 |  | 0.22 | 0.1 |  | 0 | 0.04 |  | 0.93 | 0.76 |  | 0.67 | 0.4 |
| Austria |  | 0.28 | 0 |  | 0.26 | 0.84 |  | 0 | 0 |  | 0 | 0 |  | 0 | 0 |  | 0.3 | 0.01 |
| Algeria |  | 1.57 | 1.01 |  | 0 | 0.48 |  | 0 | 0.04 |  | 0 | 3.61 |  | 0 | 0.03 |  | 4.29 | 0.69 |
| Cambodia |  | 0 | 0.13 |  | 0.07 | 0.57 |  | 0 | 0 |  | 2.11 | 1.47 |  | 0 | 0.02 |  | 0 | 0 |
| Sudan |  | 0.01 | 0 |  | 0.13 | 0.05 |  | 2.39 | 0.01 |  | 0 | 3.73 |  | 4.74 | 0.01 |  | 0.26 | 3.73 |
| Finland |  | 1.13 | 0.3 |  | 0 | 0.01 |  | 0 | 0 |  | 0 | 0 |  | 0 | 0 |  | 0.29 | 1.11 |
| Greece |  | 0.1 | 0 |  | 0.17 | 0.9 |  | 0 | 0 |  | 0.02 | 0.1 |  | 0 | 0 |  | 0.71 | 0 |
| North Korea |  | 0.05 | 0.01 |  | 0.46 | 0.05 |  | 0.03 | 0 |  | 0.5 | 1.04 |  | 0.02 | 0.02 |  | 0.06 | 0 |
| Croatia |  | 0.05 | 0 |  | 0.42 | 0.7 |  | 0 | 0 |  | 0 | 0 |  | 0 | 0.01 |  | 0.24 | 0 |
| Colombia |  | 0.01 | 0.01 |  | 0.44 | 0.06 |  | 0 | 0 |  | 0.43 | 0.63 |  | 0.08 | 0.25 |  | 0.02 | 0.03 |
| Tanzania |  | 0 | 1.78 |  | 1.29 | 0.33 |  | 0.22 | 0 |  | 0.27 | 0.2 |  | 0.45 | 0 |  | 0.07 | 0 |
| Slovakia |  | 0.17 | 0.01 |  | 0.15 | 0.63 |  | 0 | 0 |  | 0 | 0 |  | 0 | 0 |  | 0.39 | 0.08 |
| Kenya |  | 0.05 | 0.64 |  | 1.43 | 0.01 |  | 0.11 | 0 |  | 0.02 | 1.22 |  | 0.14 | 0 |  | 0.14 | 0.01 |
| Niger |  | 0 | 0 |  | 0.11 | 0.42 |  | 4.78 | 0.01 |  | 0.06 | 1.33 |  | 2.1 | 0 |  | 0.01 | 5.29 |
| New Zealand |  | 0.23 | 0.01 |  | 0.05 | 0.39 |  | 0 | 0 |  | 0 | 0 |  | 0 | 0 |  | 0.15 | 0.05 |
| Peru |  | 0.11 | 0 |  | 0.41 | 0.01 |  | 0 | 0 |  | 0.29 | 0.71 |  | 0 | 0.24 |  | 0.15 | 0 |
| Belarus |  | 0.71 | 0 |  | 0.02 | 0.9 |  | 0 | 0 |  | 0 | 0.05 |  | 0 | 0 |  | 0.35 | 0.15 |
| Afghanistan |  | 0.15 | 0.07 |  | 0.11 | 0.05 |  | 0.05 | 0 |  | 0.14 | 2.4 |  | 0 | 0.06 |  | 2.18 | 0.05 |
| Sri Lanka |  | 0 | 0 |  | 0.03 | 0.01 |  | 0.01 | 0 |  | 0.7 | 0.72 |  | 0 | 0 |  | 0 | 0 |
| Uganda |  | 0 | 0.09 |  | 0.69 | 0.31 |  | 0.41 | 0.15 |  | 0.11 | 0.9 |  | 0.3 | 0.01 |  | 0.02 | 0.07 |
| Moldova |  | 0.11 | 0.06 |  | 0.45 | 0.4 |  | 0.01 | 0 |  | 0 | 0.26 |  | 0 | 0 |  | 0.4 | 0.26 |
| Malaysia |  | 0 | 0 |  | 0.03 | 0.14 |  | 0 | 0 |  | 0.71 | 0.61 |  | 0 | 0 |  | 0 | 0 |
| Venezuela |  | 0 | 0 |  | 0.39 | 0.03 |  | 0 | 0 |  | 0.15 | 0.73 |  | 0.22 | 0.01 |  | 0 | 0 |
| Lithuania |  | 0.38 | 0.02 |  | 0 | 0.11 |  | 0 | 0 |  | 0 | 0 |  | 0 | 0 |  | 0.33 | 0.59 |
| Chile |  | 0.02 | 0 |  | 0.08 | 0.32 |  | 0 | 0 |  | 0.03 | 0 |  | 0 | 0 |  | 0.24 | 0.05 |
| Laos |  | 0 | 0.04 |  | 0.05 | 0.1 |  | 0 | 0 |  | 0.61 | 0.53 |  | 0 | 0 |  | 0 | 0 |
| Turkmenistan |  | 0.07 | 0.01 |  | 0.02 | 0.07 |  | 0 | 0 |  | 0.06 | 0.03 |  | 0 | 0 |  | 0.71 | 0.76 |
| Tunisia |  | 0.39 | 0.05 |  | 0 | 0 |  | 0 | 0 |  | 0 | 0.13 |  | 0 | 0.27 |  | 1.04 | 1 |
| Burkina Faso |  | 0 | 0 |  | 0.16 | 0.36 |  | 0.95 | 0 |  | 0.05 | 1.81 |  | 1.02 | 0 |  | 0 | 0.01 |
| Congo, DRC |  | 0 | 0.01 |  | 1.55 | 0.07 |  | 0.09 | 0.06 |  | 0.62 | 0.08 |  | 0.14 | 0.04 |  | 0 | 2.15 |
| Madagascar |  | 0 | 0 |  | 0.11 | 0 |  | 0 | 0 |  | 0.85 | 0.32 |  | 0 | 0 |  | 0 | 0.65 |
| Cote d'Ivoire |  | 0 | 0 |  | 0.7 | 0.01 |  | 0.07 | 0 |  | 0.46 | 1.29 |  | 0.06 | 0 |  | 0 | 0 |
| Uruguay |  | 0.11 | 0 |  | 0.06 | 0.01 |  | 0 | 0 |  | 0.15 | 0.47 |  | 0.03 | 0.03 |  | 0.16 | 0 |
| Mali |  | 0 | 0.02 |  | 0.14 | 0.37 |  | 0.87 | 0.02 |  | 0.26 | 1.28 |  | 0.46 | 0.03 |  | 0 | 0.01 |
| Ireland |  | 0.17 | 0 |  | 0 | 0 |  | 0 | 0 |  | 0 | 0 |  | 0 | 0 |  | 0.06 | 0.23 |
| Kyrgyzstan |  | 0.07 | 0 |  | 0.06 | 0.58 |  | 0 | 0 |  | 0.02 | 0.01 |  | 0 | 0 |  | 0.44 | 0 |
| Azerbaijan |  | 0.11 | 0 |  | 0.03 | 0.04 |  | 0 | 0 |  | 0.01 | 0.68 |  | 0 | 0 |  | 0.58 | 0.01 |
| Yemen |  | 0.11 | 0.06 |  | 0.08 | 0.76 |  | 0.21 | 0.01 |  | 0 | 0 |  | 0.84 | 0.02 |  | 0.23 | 0.63 |
| Switzerland |  | 0.07 | 0 |  | 0.02 | 0.24 |  | 0 | 0 |  | 0 | 0 |  | 0 | 0 |  | 0.14 | 0 |
| Guatemala |  | 0 | 0 |  | 0.75 | 0.03 |  | 0 | 0 |  | 0.02 | 0.7 |  | 0.04 | 0.02 |  | 0.01 | 0.08 |
| Ecuador |  | 0.04 | 0 |  | 0.4 | 0.05 |  | 0 | 0 |  | 0.26 | 0.64 |  | 0.01 | 0.06 |  | 0.03 | 0 |
| Ghana |  | 0 | 0 |  | 0.58 | 0.15 |  | 0.13 | 0 |  | 0.1 | 0.88 |  | 0.22 | 0 |  | 0 | 0 |
| Cameroon |  | 0 | 0 |  | 0.31 | 0.07 |  | 0.07 | 0.01 |  | 0.04 | 0.76 |  | 0.44 | 0.01 |  | 0 | 0.02 |
| Norway |  | 0.26 | 0 |  | 0 | 0 |  | 0 | 0 |  | 0 | 0 |  | 0 | 0 |  | 0.11 | 0.37 |
| Zimbabwe |  | 0.01 | 0.69 |  | 0.85 | 0 |  | 0.12 | 0 |  | 0 | 0 |  | 0.12 | 0 |  | 0.05 | 0.47 |
| Bolivia |  | 0.12 | 0.01 |  | 0.27 | 0.5 |  | 0 | 0 |  | 0.18 | 0.18 |  | 0.06 | 0.1 |  | 0.16 | 0 |
| Mozambique |  | 0 | 0.08 |  | 0.91 | 0.37 |  | 0.11 | 0.04 |  | 0.13 | 0.52 |  | 0.33 | 0.13 |  | 0 | 0.34 |
| Portugal |  | 0.06 | 0 |  | 0.13 | 0.29 |  | 0 | 0 |  | 0.02 | 0.09 |  | 0 | 0.02 |  | 0.19 | 0 |
| Malawi |  | 0 | 0.01 |  | 0.74 | 0.26 |  | 0.02 | 0 |  | 0.03 | 0.49 |  | 0.03 | 0 |  | 0.01 | 0.05 |
| Netherlands |  | 0.03 | 0 |  | 0.02 | 0.05 |  | 0 | 0 |  | 0 | 0 |  | 0 | 0 |  | 0.1 | 0.09 |
| Paraguay |  | 0 | 0 |  | 0.26 | 0.08 |  | 0 | 0 |  | 0.03 | 0.41 |  | 0.03 | 0 |  | 0.17 | 0 |
| Bosnia & Herzegovina |  | 0.02 | 0.02 |  | 0.19 | 0.19 |  | 0 | 0.05 |  | 0 | 0 |  | 0 | 0.02 |  | 0.08 | 0.01 |
| Cuba |  | 0 | 0 |  | 0.15 | 0 |  | 0 | 0 |  | 0.22 | 0.36 |  | 0 | 0 |  | 0 | 0 |
| Zambia |  | 0 | 0.01 |  | 0.43 | 0.03 |  | 0.09 | 0 |  | 0.03 | 0.01 |  | 0.06 | 0 |  | 0.02 | 0.58 |
| Nicaragua |  | 0 | 0 |  | 0.3 | 0.01 |  | 0 | 0 |  | 0.09 | 0.34 |  | 0.06 | 0.1 |  | 0 | 0 |
| Senegal |  | 0 | 0 |  | 0.06 | 0.28 |  | 0.54 | 0.03 |  | 0.08 | 0.47 |  | 0.12 | 0.02 |  | 0 | 0 |
| Belgium |  | 0.03 | 0.01 |  | 0.02 | 0.1 |  | 0 | 0 |  | 0 | 0 |  | 0 | 0 |  | 0.08 | 0.02 |
| Guinea |  | 0 | 0 |  | 0.07 | 0 |  | 0.02 | 0 |  | 0.37 | 0.48 |  | 0.02 | 0 |  | 0 | 0 |
| Iraq |  | 0.17 | 0 |  | 0.08 | 0.38 |  | 0 | 0 |  | 0.05 | 0.08 |  | 0 | 0 |  | 0.21 | 0.05 |
| Latvia |  | 0.13 | 0.01 |  | 0 | 0.03 |  | 0 | 0 |  | 0 | 0 |  | 0 | 0 |  | 0.14 | 0.24 |
| El Salvador |  | 0 | 0 |  | 0.23 | 0 |  | 0 | 0 |  | 0.01 | 0.32 |  | 0.09 | 0 |  | 0 | 0 |
| Chad |  | 0 | 0 |  | 0.13 | 0.02 |  | 0.63 | 0 |  | 0.02 | 0.25 |  | 0.39 | 0 |  | 0 | 0.9 |
| Macedonia |  | 0.04 | 0 |  | 0.04 | 0.1 |  | 0 | 0 |  | 0 | 0.13 |  | 0 | 0 |  | 0.15 | 0 |
| Benin |  | 0 | 0 |  | 0.34 | 0.09 |  | 0.07 | 0 |  | 0.03 | 0.48 |  | 0.15 | 0.01 |  | 0 | 0.02 |
| Slovenia |  | 0.02 | 0 |  | 0.07 | 0.1 |  | 0 | 0 |  | 0 | 0 |  | 0 | 0 |  | 0.01 | 0 |
| Togo |  | 0 | 0 |  | 0.3 | 0 |  | 0.05 | 0 |  | 0.03 | 0.56 |  | 0.19 | 0 |  | 0 | 0 |
| Dominican Republic |  | 0 | 0 |  | 0.02 | 0 |  | 0 | 0 |  | 0.12 | 0.14 |  | 0.01 | 0 |  | 0 | 0 |
| Montenegro |  | 0.01 | 0 |  | 0.12 | 0 |  | 0 | 0.22 |  | 0 | 0 |  | 0 | 0 |  | 0.08 | 0 |
| Angola |  | 0 | 0 |  | 0.68 | 0 |  | 0.24 | 0 |  | 0.01 | 0.73 |  | 0.01 | 0 |  | 0 | 0.21 |
| Honduras |  | 0 | 0 |  | 0.28 | 0.02 |  | 0 | 0 |  | 0.01 | 0.29 |  | 0.06 | 0.04 |  | 0 | 0 |
| Tajikistan |  | 0.03 | 0 |  | 0.01 | 0.03 |  | 0 | 0 |  | 0.02 | 0.01 |  | 0 | 0.26 |  | 0.24 | 0 |
| Georgia |  | 0.03 | 0 |  | 0.15 | 0.21 |  | 0 | 0 |  | 0 | 0.03 |  | 0 | 0 |  | 0.08 | 0.01 |
| Libya |  | 0.5 | 0.01 |  | 0 | 0.9 |  | 0.02 | 0.06 |  | 0 | 0 |  | 0 | 0 |  | 0.54 | 0.08 |
| Albania |  | 0 | 0 |  | 0.04 | 0.08 |  | 0 | 0.02 |  | 0 | 0 |  | 0 | 0 |  | 0.1 | 0.04 |
| Haiti |  | 0 | 0 |  | 0.27 | 0 |  | 0 | 0 |  | 0.06 | 0.43 |  | 0.1 | 0 |  | 0 | 0 |
| Somalia |  | 0 | 0 |  | 0.19 | 0.01 |  | 0 | 0 |  | 0 | 0.39 |  | 0.21 | 0 |  | 0 | 0 |
| Guyana |  | 0 | 0 |  | 0.01 | 0.01 |  | 0 | 0 |  | 0.11 | 0.1 |  | 0 | 0 |  | 0 | 0 |
| Suriname |  | 0 | 0 |  | 0 | 0 |  | 0 | 0 |  | 0.09 | 0.09 |  | 0 | 0 |  | 0 | 0 |
| Estonia |  | 0.11 | 0.08 |  | 0 | 0.01 |  | 0 | 0 |  | 0 | 0 |  | 0 | 0 |  | 0.05 | 0.07 |
| Liberia |  | 0 | 0 |  | 0.01 | 0.01 |  | 0 | 0 |  | 0.25 | 0.25 |  | 0 | 0 |  | 0 | 0 |
| Sierra Leone |  | 0 | 0 |  | 0.01 | 0.03 |  | 0.01 | 0.03 |  | 0.22 | 0.2 |  | 0.01 | 0 |  | 0 | 0 |
| Lebanon |  | 0.04 | 0 |  | 0 | 0.07 |  | 0 | 0 |  | 0 | 0 |  | 0 | 0 |  | 0.07 | 0.05 |
| Armenia |  | 0.06 | 0 |  | 0 | 0.12 |  | 0 | 0 |  | 0 | 0.05 |  | 0 | 0 |  | 0.1 | 0 |
| Burundi |  | 0 | 0.02 |  | 0.1 | 0.02 |  | 0.01 | 0.01 |  | 0.01 | 0.11 |  | 0.04 | 0.01 |  | 0.01 | 0 |
| Costa Rica |  | 0 | 0 |  | 0.01 | 0 |  | 0 | 0 |  | 0.05 | 0.07 |  | 0 | 0 |  | 0 | 0 |
| Panama |  | 0 | 0 |  | 0.04 | 0.02 |  | 0 | 0 |  | 0.07 | 0.05 |  | 0 | 0.05 |  | 0 | 0 |
| Central African Republic |  | 0 | 0 |  | 0.12 | 0 |  | 0.02 | 0 |  | 0.03 | 0.2 |  | 0.06 | 0 |  | 0 | 0.02 |
| Mongolia |  | 0.01 | 0.15 |  | 0.01 | 0.05 |  | 0 | 0 |  | 0 | 0 |  | 0 | 0.01 |  | 0.22 | 0.02 |
| Rwanda |  | 0 | 0.01 |  | 0.07 | 0.01 |  | 0 | 0 |  | 0.01 | 0.17 |  | 0.11 | 0.01 |  | 0.01 | 0.01 |
| Israel |  | 0.03 | 0 |  | 0.01 | 0.05 |  | 0 | 0 |  | 0 | 0 |  | 0 | 0.02 |  | 0.04 | 0 |
| The Gambia |  | 0 | 0 |  | 0.01 | 0.03 |  | 0.11 | 0 |  | 0.02 | 0.13 |  | 0.01 | 0 |  | 0 | 0 |
| Eritrea |  | 0.04 | 0.01 |  | 0.02 | 0.04 |  | 0.05 | 0 |  | 0 | 0.1 |  | 0.18 | 0.11 |  | 0.02 | 0.06 |
| Lesotho |  | 0 | 0 |  | 0.1 | 0.01 |  | 0 | 0 |  | 0 | 0.02 |  | 0.02 | 0.06 |  | 0.01 | 0.04 |
| Jordan |  | 0.07 | 0 |  | 0 | 0.09 |  | 0 | 0 |  | 0 | 0 |  | 0 | 0 |  | 0.02 | 0 |
| Timor-Leste |  | 0 | 0 |  | 0.06 | 0 |  | 0 | 0 |  | 0.02 | 0.07 |  | 0 | 0 |  | 0 | 0 |
| Mauritania |  | 0 | 0.08 |  | 0.02 | 0 |  | 0.02 | 0 |  | 0.02 | 0.1 |  | 0.13 | 0 |  | 0 | 0 |
| Bhutan |  | 0 | 0 |  | 0.02 | 0.02 |  | 0.01 | 0 |  | 0.04 | 0.04 |  | 0 | 0 |  | 0.01 | 0.01 |
| Guinea-Bissau |  | 0 | 0 |  | 0.02 | 0 |  | 0.02 | 0 |  | 0.05 | 0.1 |  | 0.01 | 0 |  | 0 | 0 |
| Swaziland |  | 0 | 0 |  | 0.06 | 0 |  | 0 | 0 |  | 0 | 0.06 |  | 0 | 0 |  | 0 | 0 |
| Namibia |  | 0 | 0 |  | 0.05 | 0 |  | 0.13 | 0 |  | 0 | 0 |  | 0.02 | 0 |  | 0 | 0.2 |
| Botswana |  | 0 | 0.02 |  | 0.08 | 0.01 |  | 0.01 | 0 |  | 0 | 0.02 |  | 0.05 | 0 |  | 0 | 0.1 |
| West Bank |  | 0 | 0 |  | 0 | 0.01 |  | 0 | 0 |  | 0 | 0 |  | 0 | 0.01 |  | 0.02 | 0 |
| Gabon |  | 0 | 0 |  | 0.04 | 0.01 |  | 0 | 0 |  | 0 | 0.03 |  | 0 | 0 |  | 0 | 0 |
| Belize |  | 0 | 0 |  | 0.01 | 0 |  | 0 | 0 |  | 0 | 0 |  | 0 | 0.01 |  | 0 | 0 |
| Luxembourg |  | 0 | 0 |  | 0 | 0 |  | 0 | 0 |  | 0 | 0 |  | 0 | 0 |  | 0 | 0 |
| Oman |  | 0 | 0 |  | 0 | 0 |  | 0 | 0 |  | 0 | 0 |  | 0.01 | 0 |  | 0 | 0.01 |
| French Guiana |  | 0 | 0 |  | 0 | 0 |  | 0 | 0 |  | 0.01 | 0.01 |  | 0 | 0 |  | 0 | 0 |
| Kuwait |  | 0.01 | 0 |  | 0 | 0.01 |  | 0 | 0 |  | 0 | 0 |  | 0 | 0 |  | 0 | 0 |
| Congo |  | 0 | 0 |  | 0.03 | 0.03 |  | 0 | 0 |  | 0.01 | 0.01 |  | 0 | 0 |  | 0 | 0.01 |
| Trinidad & Tobago |  | 0 | 0 |  | 0 | 0 |  | 0 | 0 |  | 0 | 0 |  | 0 | 0 |  | 0 | 0 |
| United Arab Emirates |  | 0 | 0 |  | 0 | 0 |  | 0 | 0 |  | 0 | 0 |  | 0 | 0 |  | 0 | 0 |
| Liechtenstein |  | 0 | 0 |  | 0 | 0 |  | 0 | 0 |  | 0 | 0 |  | 0 | 0 |  | 0 | 0 |
| Equatorial Guinea |  | 0 | 0 |  | 0 | 0 |  | 0 | 0 |  | 0 | 0.01 |  | 0.01 | 0 |  | 0 | 0 |
| Papua New Guinea |  | 0 | 0 |  | 0 | 0 |  | 0 | 0 |  | 0 | 0 |  | 0 | 0 |  | 0 | 0 |
| San Marino |  | 0 | 0 |  | 0 | 0 |  | 0 | 0 |  | 0 | 0 |  | 0 | 0 |  | 0 | 0 |
| Jamaica |  | 0 | 0 |  | 0 | 0 |  | 0 | 0 |  | 0 | 0 |  | 0 | 0 |  | 0 | 0 |
| Andorra |  | 0 | 0 |  | 0 | 0 |  | 0 | 0 |  | 0 | 0 |  | 0 | 0 |  | 0 | 0 |
| Gaza Strip |  | 0 | 0 |  | 0 | 0 |  | 0 | 0 |  | 0 | 0 |  | 0 | 0 |  | 0 | 0 |
| Puerto Rico |  | 0 | 0 |  | 0 | 0 |  | 0 | 0 |  | 0 | 0 |  | 0 | 0 |  | 0 | 0 |
| Brunei |  | 0 | 0 |  | 0 | 0 |  | 0 | 0 |  | 0 | 0 |  | 0 | 0 |  | 0 | 0 |
| Bahrain |  | 0 | 0 |  | 0 | 0 |  | 0 | 0 |  | 0 | 0 |  | 0 | 0 |  | 0 | 0 |
| Singapore |  | 0 | 0 |  | 0 | 0 |  | 0 | 0 |  | 0 | 0 |  | 0 | 0 |  | 0 | 0 |
| Isle of Man |  | 0 | 0 |  | 0 | 0 |  | 0 | 0 |  | 0 | 0 |  | 0 | 0 |  | 0 | 0 |
| Vatican City |  | 0 | 0 |  | 0 | 0 |  | 0 | 0 |  | 0 | 0 |  | 0 | 0 |  | 0 | 0 |
| Monaco |  | 0 | 0 |  | 0 | 0 |  | 0 | 0 |  | 0 | 0 |  | 0 | 0 |  | 0 | 0 |
| Gibraltar |  | 0 | 0 |  | 0 | 0 |  | 0 | 0 |  | 0 | 0 |  | 0 | 0 |  | 0 | 0 |
| Jersey |  | 0 | 0 |  | 0 | 0 |  | 0 | 0 |  | 0 | 0 |  | 0 | 0 |  | 0 | 0 |
