# Supplementary material for: Transformative optimisation of agricultural land use to meet future food demands
Source: PeerJ. 2013 Oct 24;1:e188. doi: 10.7717/peerj.188 (PMC3817586; doi:10.7717/peerj.188)
Supplement: Table S2 [file peerj-01-188-s004.docx]

**Table S2. Harvested area of oilseed crops in 167 countries under current and optimal land-use allocation options (all values in 10^6^ ha).**

|  |  | Soy | |  | Cottonseed | |  | Rapeseed | |  | Sunflower seed | |  | Groundnut | |  | Oil palm | |
| --- | --- | --- | --- | --- | --- | --- | --- | --- | --- | --- | --- | --- | --- | --- | --- | --- | --- | --- |
| Country |  | Current | Optimal |  | Current | Optimal |  | Current | Optimal |  | Current | Optimal |  | Current | Optimal |  | Current | Optimal |
| United States |  | 29.69 | 34.98 |  | 5.18 | 0.74 |  | 0.49 | 0.48 |  | 1.08 | 0.15 |  | 0.53 | 0.62 |  | 0 | 0 |
| Malaysia |  | 0 | 0 |  | 0 | 0 |  | 0 | 0 |  | 0 | 0 |  | 0 | 0 |  | 2.88 | 2.88 |
| China |  | 8.11 | 17.08 |  | 4.41 | 4.29 |  | 6.95 | 2.52 |  | 1.01 | 0.94 |  | 4.39 | 0.03 |  | 0 | 0 |
| Indonesia |  | 0.7 | 0.74 |  | 0 | 0 |  | 0 | 0 |  | 0 | 0 |  | 0.63 | 0 |  | 2.24 | 2.83 |
| Brazil |  | 13.15 | 13.81 |  | 0.69 | 0.02 |  | 0 | 0.27 |  | 0.1 | 0 |  | 0.12 | 0 |  | 0.04 | 0.01 |
| Argentina |  | 8.82 | 12.16 |  | 0.44 | 0.02 |  | 0 | 0 |  | 2.65 | 0 |  | 0.27 | 0 |  | 0 | 0 |
| India |  | 6.09 | 15.59 |  | 7.13 | 0.26 |  | 5.52 | 8.5 |  | 1.55 | 2.01 |  | 6.35 | 0.29 |  | 0 | 0 |
| Nigeria |  | 0.52 | 5.39 |  | 0.47 | 0.28 |  | 0 | 0 |  | 0 | 0 |  | 2.28 | 0.41 |  | 2.83 | 0.02 |
| Canada |  | 1.39 | 5.08 |  | 0 | 0 |  | 5.06 | 1.4 |  | 0.09 | 0.06 |  | 0 | 0 |  | 0 | 0 |
| Pakistan |  | 0 | 3.66 |  | 3.12 | 0 |  | 0.37 | 0.06 |  | 0.13 | 0.01 |  | 0.11 | 0 |  | 0 | 0 |
| Australia |  | 0.14 | 0.98 |  | 0.85 | 1.22 |  | 1.79 | 0.7 |  | 0.17 | 0.07 |  | 0.04 | 0.02 |  | 0 | 0 |
| France |  | 0.12 | 0.66 |  | 0 | 0 |  | 1.07 | 1.29 |  | 0.75 | 0 |  | 0 | 0 |  | 0 | 0 |
| Russia |  | 0.58 | 4.43 |  | 0.02 | 0.09 |  | 0.32 | 0.03 |  | 4.37 | 0.74 |  | 0 | 0 |  | 0 | 0 |
| Thailand |  | 0.2 | 0.07 |  | 0.04 | 0 |  | 0 | 0 |  | 0.05 | 0 |  | 0.08 | 0 |  | 0.23 | 0.52 |
| Uzbekistan |  | 0 | 0.03 |  | 1.58 | 0.61 |  | 0 | 0.02 |  | 0.02 | 0.94 |  | 0.01 | 0 |  | 0 | 0 |
| Ukraine |  | 0.09 | 3.16 |  | 0 | 0 |  | 0.11 | 0.01 |  | 2.98 | 0 |  | 0 | 0 |  | 0 | 0 |
| Turkey |  | 0.03 | 0.4 |  | 0.73 | 0.02 |  | 0 | 0.86 |  | 0.49 | 0.01 |  | 0.03 | 0 |  | 0 | 0 |
| Germany |  | 0 | 0.04 |  | 0 | 0 |  | 0.96 | 0.95 |  | 0.04 | 0 |  | 0 | 0 |  | 0 | 0 |
| Paraguay |  | 0.97 | 1.21 |  | 0.15 | 0 |  | 0 | 0 |  | 0.04 | 0 |  | 0.03 | 0 |  | 0.01 | 0 |
| Colombia |  | 0.04 | 0.07 |  | 0.07 | 0.02 |  | 0 | 0 |  | 0 | 0 |  | 0 | 0 |  | 0.14 | 0.16 |
| Others (below) |  | 2.98 | 13.74 |  | 7.22 | 5.85 |  | 2.71 | 3.13 |  | 5.76 | 1.6 |  | 7.35 | 1.09 |  | 1.46 | 2.08 |
| Syria |  | 0.01 | 0.1 |  | 0.51 | 0.37 |  | 0 | 0.01 |  | 0.01 | 0.07 |  | 0.02 | 0 |  | 0 | 0 |
| Cote d'Ivoire |  | 0 | 0.14 |  | 0.28 | 0.26 |  | 0 | 0 |  | 0 | 0 |  | 0.15 | 0 |  | 0.14 | 0.17 |
| United Kingdom |  | 0 | 0 |  | 0 | 0 |  | 0.49 | 0.49 |  | 0 | 0 |  | 0 | 0 |  | 0 | 0 |
| Ecuador |  | 0.05 | 0.01 |  | 0.01 | 0 |  | 0 | 0 |  | 0 | 0 |  | 0.02 | 0 |  | 0.12 | 0.18 |
| Italy |  | 0.28 | 0.4 |  | 0 | 0 |  | 0.04 | 0.06 |  | 0.15 | 0.01 |  | 0 | 0 |  | 0 | 0 |
| Congo, DRC |  | 0.03 | 0.32 |  | 0.11 | 0.02 |  | 0 | 0 |  | 0 | 0.01 |  | 0.57 | 0.11 |  | 0.27 | 0.53 |
| Bolivia |  | 0.58 | 0.81 |  | 0.08 | 0 |  | 0 | 0 |  | 0.13 | 0 |  | 0.02 | 0 |  | 0 | 0 |
| Ghana |  | 0 | 0.02 |  | 0.04 | 0.12 |  | 0 | 0 |  | 0 | 0 |  | 0.2 | 0.05 |  | 0.12 | 0.17 |
| Spain |  | 0 | 0.84 |  | 0.11 | 0.01 |  | 0.04 | 0.15 |  | 0.85 | 0 |  | 0 | 0 |  | 0 | 0 |
| Sudan |  | 0 | 0.03 |  | 0.2 | 1.53 |  | 0 | 0 |  | 0.01 | 0.12 |  | 1.47 | 0 |  | 0 | 0 |
| Romania |  | 0.09 | 0.99 |  | 0 | 0 |  | 0.05 | 0.06 |  | 0.94 | 0.03 |  | 0 | 0 |  | 0 | 0 |
| Myanmar |  | 0.11 | 1.01 |  | 0.29 | 0.04 |  | 0.01 | 0.02 |  | 0.35 | 0.18 |  | 0.52 | 0.01 |  | 0 | 0.01 |
| Egypt |  | 0.02 | 0.5 |  | 0.4 | 0 |  | 0 | 0 |  | 0.03 | 0.02 |  | 0.09 | 0 |  | 0 | 0 |
| South Africa |  | 0.13 | 0.87 |  | 0.08 | 0 |  | 0 | 0 |  | 0.55 | 0 |  | 0.11 | 0 |  | 0 | 0 |
| Cameroon |  | 0.02 | 0.33 |  | 0.2 | 0.11 |  | 0 | 0 |  | 0 | 0 |  | 0.28 | 0 |  | 0.05 | 0.12 |
| Turkmenistan |  | 0 | 0.03 |  | 0.69 | 0.6 |  | 0 | 0.03 |  | 0 | 0.03 |  | 0 | 0.01 |  | 0 | 0 |
| Greece |  | 0 | 0.26 |  | 0.33 | 0.09 |  | 0 | 0 |  | 0.03 | 0.01 |  | 0 | 0 |  | 0 | 0 |
| Iran |  | 0.16 | 0.63 |  | 0.36 | 0.02 |  | 0 | 0 |  | 0.15 | 0.01 |  | 0 | 0.01 |  | 0 | 0 |
| Poland |  | 0 | 0 |  | 0 | 0 |  | 0.45 | 0.46 |  | 0.01 | 0 |  | 0 | 0 |  | 0 | 0 |
| Guinea |  | 0 | 0.01 |  | 0.04 | 0.46 |  | 0 | 0 |  | 0 | 0 |  | 0.15 | 0 |  | 0.28 | 0.01 |
| Hungary |  | 0.02 | 0.4 |  | 0 | 0 |  | 0.11 | 0.11 |  | 0.4 | 0.03 |  | 0 | 0 |  | 0 | 0 |
| Mexico |  | 0.08 | 0.09 |  | 0.11 | 0.08 |  | 0 | 0.09 |  | 0 | 0.01 |  | 0.09 | 0 |  | 0.01 | 0.02 |
| Papua New Guinea |  | 0 | 0 |  | 0 | 0 |  | 0 | 0 |  | 0 | 0 |  | 0 | 0.03 |  | 0.08 | 0.04 |
| Czech Republic |  | 0.01 | 0 |  | 0 | 0 |  | 0.25 | 0.27 |  | 0.02 | 0 |  | 0 | 0 |  | 0 | 0 |
| Honduras |  | 0 | 0.01 |  | 0 | 0 |  | 0 | 0 |  | 0 | 0 |  | 0 | 0 |  | 0.04 | 0.04 |
| Bulgaria |  | 0.01 | 0.24 |  | 0.01 | 0 |  | 0.01 | 0.34 |  | 0.53 | 0 |  | 0.01 | 0 |  | 0 | 0 |
| Senegal |  | 0 | 0 |  | 0.02 | 0.32 |  | 0 | 0 |  | 0 | 0 |  | 0.59 | 0.12 |  | 0.01 | 0.18 |
| Kazakhstan |  | 0.02 | 0.52 |  | 0.21 | 0.04 |  | 0.02 | 0 |  | 0.31 | 0 |  | 0 | 0 |  | 0 | 0 |
| Costa Rica |  | 0 | 0 |  | 0 | 0 |  | 0 | 0 |  | 0 | 0 |  | 0 | 0 |  | 0.03 | 0.03 |
| Benin |  | 0.01 | 0.25 |  | 0.28 | 0.01 |  | 0 | 0 |  | 0 | 0 |  | 0.08 | 0 |  | 0.02 | 0.14 |
| Serbia |  | 0.1 | 0.17 |  | 0 | 0 |  | 0.01 | 0.03 |  | 0.17 | 0.07 |  | 0 | 0 |  | 0 | 0 |
| Vietnam |  | 0.13 | 0.3 |  | 0.03 | 0.05 |  | 0 | 0 |  | 0 | 0.01 |  | 0.2 | 0.02 |  | 0 | 0 |
| Uganda |  | 0.18 | 0.56 |  | 0.25 | 0 |  | 0 | 0 |  | 0.02 | 0.08 |  | 0.2 | 0 |  | 0 | 0 |
| Guatemala |  | 0.01 | 0.01 |  | 0 | 0 |  | 0 | 0 |  | 0 | 0 |  | 0 | 0 |  | 0.02 | 0.03 |
| Zimbabwe |  | 0.06 | 0.46 |  | 0.25 | 0 |  | 0 | 0 |  | 0.04 | 0.05 |  | 0.16 | 0 |  | 0 | 0 |
| Tajikistan |  | 0 | 0 |  | 0.22 | 0.19 |  | 0 | 0 |  | 0 | 0.03 |  | 0 | 0 |  | 0 | 0 |
| North Korea |  | 0.29 | 0.3 |  | 0.02 | 0 |  | 0 | 0 |  | 0 | 0 |  | 0 | 0 |  | 0 | 0 |
| Burkina Faso |  | 0 | 0.37 |  | 0.17 | 0 |  | 0 | 0 |  | 0 | 0 |  | 0.2 | 0 |  | 0 | 0 |
| Denmark |  | 0 | 0 |  | 0 | 0 |  | 0.12 | 0.12 |  | 0 | 0 |  | 0 | 0 |  | 0 | 0 |
| Bangladesh |  | 0 | 0.01 |  | 0.02 | 0.27 |  | 0.35 | 0.12 |  | 0 | 0 |  | 0.03 | 0 |  | 0 | 0 |
| Austria |  | 0.02 | 0.04 |  | 0 | 0 |  | 0.07 | 0.09 |  | 0.03 | 0 |  | 0 | 0 |  | 0 | 0 |
| Mali |  | 0 | 0.03 |  | 0.18 | 0.19 |  | 0 | 0 |  | 0 | 0 |  | 0.15 | 0.11 |  | 0 | 0.01 |
| Moldova |  | 0.01 | 0.24 |  | 0 | 0 |  | 0 | 0 |  | 0.24 | 0.01 |  | 0 | 0 |  | 0 | 0 |
| Slovakia |  | 0.01 | 0.05 |  | 0 | 0 |  | 0.08 | 0.1 |  | 0.07 | 0.01 |  | 0 | 0 |  | 0 | 0 |
| Chad |  | 0 | 0.06 |  | 0.14 | 0.19 |  | 0 | 0 |  | 0 | 0 |  | 0.21 | 0.09 |  | 0 | 0 |
| Togo |  | 0 | 0.03 |  | 0.17 | 0.09 |  | 0 | 0 |  | 0 | 0 |  | 0.05 | 0.01 |  | 0.01 | 0.11 |
| Tanzania |  | 0 | 0.47 |  | 0.3 | 0.01 |  | 0 | 0 |  | 0.11 | 0.06 |  | 0.13 | 0 |  | 0 | 0 |
| Liberia |  | 0.01 | 0.01 |  | 0 | 0.01 |  | 0 | 0 |  | 0 | 0 |  | 0.01 | 0 |  | 0.02 | 0.04 |
| Singapore |  | 0 | 0 |  | 0 | 0 |  | 0 | 0 |  | 0 | 0 |  | 0 | 0 |  | 0.01 | 0.01 |
| Japan |  | 0.11 | 0.05 |  | 0 | 0 |  | 0 | 0.07 |  | 0 | 0 |  | 0.01 | 0 |  | 0 | 0 |
| Sierra Leone |  | 0 | 0 |  | 0 | 0.01 |  | 0 | 0 |  | 0 | 0 |  | 0.03 | 0.01 |  | 0.03 | 0.04 |
| Mozambique |  | 0 | 0.04 |  | 0.13 | 0.02 |  | 0 | 0 |  | 0.05 | 0.37 |  | 0.26 | 0 |  | 0 | 0 |
| Finland |  | 0 | 0 |  | 0 | 0 |  | 0.13 | 0.13 |  | 0 | 0 |  | 0 | 0 |  | 0 | 0 |
| Croatia |  | 0.04 | 0.07 |  | 0 | 0 |  | 0.02 | 0.02 |  | 0.03 | 0 |  | 0 | 0 |  | 0 | 0 |
| Peru |  | 0 | 0.11 |  | 0.1 | 0 |  | 0 | 0 |  | 0 | 0 |  | 0 | 0 |  | 0.01 | 0 |
| Afghanistan |  | 0 | 0.01 |  | 0.12 | 0.09 |  | 0 | 0 |  | 0 | 0.03 |  | 0 | 0 |  | 0 | 0 |
| Zambia |  | 0.01 | 0.23 |  | 0.07 | 0 |  | 0 | 0 |  | 0.03 | 0 |  | 0.12 | 0 |  | 0 | 0 |
| Central African Republic |  | 0 | 0.02 |  | 0.05 | 0.05 |  | 0 | 0 |  | 0 | 0 |  | 0.12 | 0.1 |  | 0 | 0 |
| Venezuela |  | 0 | 0.06 |  | 0.03 | 0 |  | 0 | 0 |  | 0 | 0 |  | 0 | 0 |  | 0.03 | 0 |
| Niger |  | 0 | 0.05 |  | 0.04 | 0.06 |  | 0 | 0 |  | 0 | 0 |  | 0.21 | 0.14 |  | 0 | 0 |
| Kyrgyzstan |  | 0 | 0 |  | 0.04 | 0.06 |  | 0 | 0 |  | 0.04 | 0.02 |  | 0 | 0 |  | 0 | 0 |
| Sweden |  | 0 | 0 |  | 0 | 0 |  | 0.06 | 0.06 |  | 0 | 0 |  | 0 | 0 |  | 0 | 0 |
| The Gambia |  | 0 | 0 |  | 0 | 0 |  | 0 | 0 |  | 0 | 0 |  | 0.1 | 0.01 |  | 0 | 0.1 |
| Uruguay |  | 0.02 | 0.1 |  | 0 | 0 |  | 0 | 0 |  | 0.08 | 0.01 |  | 0 | 0 |  | 0 | 0 |
| Ethiopia |  | 0 | 0.14 |  | 0.07 | 0 |  | 0.03 | 0 |  | 0 | 0 |  | 0.04 | 0 |  | 0 | 0 |
| South Korea |  | 0.08 | 0.09 |  | 0 | 0 |  | 0 | 0 |  | 0 | 0 |  | 0.01 | 0 |  | 0 | 0 |
| Solomon Is. |  | 0 | 0 |  | 0 | 0 |  | 0 | 0 |  | 0 | 0 |  | 0 | 0 |  | 0.01 | 0.01 |
| Morocco |  | 0 | 0.11 |  | 0 | 0.04 |  | 0 | 0 |  | 0.12 | 0 |  | 0.03 | 0 |  | 0 | 0 |
| Nicaragua |  | 0.01 | 0.04 |  | 0 | 0 |  | 0 | 0 |  | 0 | 0 |  | 0.02 | 0 |  | 0 | 0 |
| Azerbaijan |  | 0.01 | 0.1 |  | 0.1 | 0 |  | 0 | 0 |  | 0.01 | 0 |  | 0 | 0.01 |  | 0 | 0 |
| Dominican Republic |  | 0 | 0 |  | 0 | 0 |  | 0 | 0 |  | 0 | 0 |  | 0 | 0 |  | 0.01 | 0.01 |
| Malawi |  | 0 | 0.02 |  | 0.04 | 0.06 |  | 0 | 0 |  | 0.01 | 0.07 |  | 0.09 | 0 |  | 0 | 0 |
| Equatorial Guinea |  | 0 | 0 |  | 0 | 0 |  | 0 | 0 |  | 0 | 0 |  | 0 | 0 |  | 0.01 | 0.01 |
| Belarus |  | 0 | 0.01 |  | 0 | 0 |  | 0.08 | 0.02 |  | 0.02 | 0.08 |  | 0 | 0 |  | 0 | 0 |
| Laos |  | 0.01 | 0 |  | 0.01 | 0.04 |  | 0 | 0 |  | 0 | 0 |  | 0.02 | 0 |  | 0 | 0.01 |
| Cambodia |  | 0.05 | 0.05 |  | 0 | 0.02 |  | 0 | 0 |  | 0 | 0 |  | 0.02 | 0 |  | 0 | 0 |
| Kenya |  | 0.01 | 0.02 |  | 0.06 | 0 |  | 0 | 0 |  | 0.02 | 0.09 |  | 0.03 | 0 |  | 0 | 0 |
| Madagascar |  | 0 | 0.09 |  | 0.04 | 0 |  | 0 | 0 |  | 0 | 0 |  | 0.06 | 0 |  | 0 | 0 |
| Guinea-Bissau |  | 0 | 0 |  | 0 | 0.02 |  | 0 | 0 |  | 0 | 0 |  | 0.01 | 0 |  | 0.01 | 0.01 |
| Israel |  | 0 | 0 |  | 0.02 | 0.02 |  | 0 | 0 |  | 0 | 0 |  | 0 | 0 |  | 0 | 0 |
| Gabon |  | 0.01 | 0.04 |  | 0 | 0 |  | 0 | 0 |  | 0 | 0 |  | 0.05 | 0 |  | 0.01 | 0.02 |
| Algeria |  | 0 | 0 |  | 0 | 0 |  | 0.03 | 0.04 |  | 0 | 0 |  | 0.01 | 0 |  | 0 | 0 |
| Congo |  | 0 | 0.02 |  | 0 | 0.01 |  | 0 | 0 |  | 0 | 0 |  | 0.07 | 0.06 |  | 0.01 | 0 |
| Switzerland |  | 0.01 | 0.02 |  | 0 | 0 |  | 0.01 | 0.01 |  | 0 | 0 |  | 0 | 0 |  | 0 | 0 |
| Chile |  | 0 | 0 |  | 0 | 0 |  | 0.02 | 0.03 |  | 0 | 0 |  | 0 | 0 |  | 0 | 0 |
| Lithuania |  | 0 | 0 |  | 0 | 0 |  | 0.04 | 0.04 |  | 0 | 0 |  | 0 | 0 |  | 0 | 0 |
| Angola |  | 0 | 0.01 |  | 0.01 | 0.12 |  | 0 | 0 |  | 0.01 | 0.02 |  | 0.11 | 0 |  | 0.03 | 0 |
| Lebanon |  | 0 | 0 |  | 0.01 | 0.01 |  | 0 | 0 |  | 0 | 0 |  | 0 | 0 |  | 0 | 0 |
| Jordan |  | 0 | 0 |  | 0.01 | 0.01 |  | 0 | 0 |  | 0 | 0 |  | 0 | 0 |  | 0 | 0 |
| Montenegro |  | 0.01 | 0.02 |  | 0 | 0 |  | 0 | 0 |  | 0.02 | 0 |  | 0 | 0 |  | 0 | 0 |
| Sao Tome & Principe |  | 0 | 0 |  | 0 | 0 |  | 0 | 0 |  | 0 | 0 |  | 0 | 0 |  | 0 | 0 |
| Yemen |  | 0 | 0 |  | 0.04 | 0.04 |  | 0 | 0 |  | 0 | 0 |  | 0 | 0 |  | 0 | 0 |
| Libya |  | 0 | 0 |  | 0 | 0 |  | 0 | 0 |  | 0 | 0 |  | 0.02 | 0.02 |  | 0 | 0 |
| Nepal |  | 0.02 | 0.04 |  | 0 | 0.01 |  | 0.03 | 0.01 |  | 0 | 0 |  | 0 | 0 |  | 0 | 0 |
| Philippines |  | 0 | 0.03 |  | 0 | 0.01 |  | 0 | 0 |  | 0 | 0 |  | 0.03 | 0.02 |  | 0.02 | 0 |
| Georgia |  | 0 | 0.04 |  | 0.01 | 0 |  | 0 | 0 |  | 0.03 | 0 |  | 0 | 0 |  | 0 | 0 |
| Iraq |  | 0 | 0 |  | 0.01 | 0 |  | 0 | 0 |  | 0 | 0.01 |  | 0 | 0 |  | 0 | 0 |
| Haiti |  | 0 | 0 |  | 0 | 0 |  | 0 | 0 |  | 0 | 0 |  | 0.03 | 0.03 |  | 0 | 0 |
| West Bank |  | 0 | 0 |  | 0.01 | 0.01 |  | 0 | 0 |  | 0 | 0 |  | 0 | 0 |  | 0 | 0 |
| El Salvador |  | 0 | 0 |  | 0 | 0 |  | 0 | 0 |  | 0 | 0 |  | 0 | 0 |  | 0 | 0 |
| Panama |  | 0 | 0 |  | 0 | 0 |  | 0 | 0 |  | 0 | 0 |  | 0 | 0 |  | 0.01 | 0 |
| Brunei |  | 0 | 0 |  | 0 | 0 |  | 0 | 0 |  | 0 | 0 |  | 0 | 0 |  | 0 | 0 |
| Portugal |  | 0 | 0.01 |  | 0 | 0 |  | 0 | 0.01 |  | 0.05 | 0 |  | 0 | 0.03 |  | 0 | 0 |
| Rwanda |  | 0.03 | 0.04 |  | 0.01 | 0 |  | 0 | 0 |  | 0 | 0 |  | 0.01 | 0 |  | 0 | 0 |
| Estonia |  | 0 | 0 |  | 0 | 0 |  | 0.02 | 0.02 |  | 0 | 0 |  | 0 | 0 |  | 0 | 0 |
| Bosnia & Herzegovina |  | 0.01 | 0.01 |  | 0 | 0 |  | 0 | 0.02 |  | 0.02 | 0 |  | 0 | 0 |  | 0 | 0 |
| Macedonia |  | 0 | 0.01 |  | 0.01 | 0 |  | 0 | 0.01 |  | 0.01 | 0 |  | 0 | 0 |  | 0 | 0 |
| Norway |  | 0 | 0 |  | 0 | 0 |  | 0.01 | 0.01 |  | 0 | 0 |  | 0 | 0 |  | 0 | 0 |
| Botswana |  | 0 | 0.01 |  | 0 | 0.01 |  | 0 | 0 |  | 0.01 | 0 |  | 0.01 | 0 |  | 0 | 0 |
| Burundi |  | 0.01 | 0.03 |  | 0 | 0 |  | 0 | 0 |  | 0 | 0 |  | 0.01 | 0 |  | 0 | 0 |
| Tunisia |  | 0 | 0 |  | 0 | 0.02 |  | 0 | 0 |  | 0.01 | 0 |  | 0 | 0 |  | 0 | 0 |
| Swaziland |  | 0 | 0.01 |  | 0.01 | 0 |  | 0 | 0 |  | 0 | 0 |  | 0.01 | 0.01 |  | 0 | 0 |
| New Zealand |  | 0 | 0 |  | 0 | 0 |  | 0.01 | 0.01 |  | 0 | 0 |  | 0 | 0 |  | 0 | 0 |
| Belgium |  | 0 | 0 |  | 0 | 0 |  | 0.01 | 0 |  | 0 | 0 |  | 0 | 0 |  | 0 | 0 |
| Latvia |  | 0 | 0 |  | 0 | 0 |  | 0.01 | 0.01 |  | 0 | 0 |  | 0 | 0 |  | 0 | 0 |
| Bhutan |  | 0 | 0.02 |  | 0 | 0 |  | 0.03 | 0.01 |  | 0 | 0 |  | 0 | 0 |  | 0 | 0 |
| Slovenia |  | 0 | 0.01 |  | 0 | 0 |  | 0 | 0 |  | 0 | 0 |  | 0 | 0 |  | 0 | 0 |
| Netherlands |  | 0 | 0 |  | 0 | 0 |  | 0.01 | 0 |  | 0 | 0 |  | 0 | 0 |  | 0 | 0 |
| Cuba |  | 0 | 0 |  | 0 | 0 |  | 0 | 0 |  | 0 | 0 |  | 0.01 | 0.01 |  | 0 | 0 |
| Armenia |  | 0 | 0.01 |  | 0.01 | 0 |  | 0 | 0 |  | 0 | 0 |  | 0 | 0 |  | 0 | 0 |
| Ireland |  | 0 | 0 |  | 0 | 0 |  | 0 | 0 |  | 0 | 0 |  | 0 | 0 |  | 0 | 0 |
| Albania |  | 0 | 0.01 |  | 0 | 0 |  | 0 | 0 |  | 0 | 0 |  | 0 | 0 |  | 0 | 0 |
| Lesotho |  | 0 | 0.01 |  | 0 | 0 |  | 0 | 0 |  | 0.01 | 0 |  | 0 | 0 |  | 0 | 0 |
| Sri Lanka |  | 0 | 0.01 |  | 0 | 0 |  | 0 | 0 |  | 0 | 0 |  | 0.01 | 0 |  | 0 | 0 |
| Timor-Leste |  | 0 | 0.01 |  | 0 | 0 |  | 0 | 0 |  | 0 | 0 |  | 0 | 0 |  | 0 | 0 |
| Eritrea |  | 0 | 0 |  | 0 | 0 |  | 0 | 0 |  | 0 | 0 |  | 0 | 0 |  | 0 | 0 |
| Luxembourg |  | 0 | 0 |  | 0 | 0 |  | 0 | 0 |  | 0 | 0 |  | 0 | 0 |  | 0 | 0 |
| Guyana |  | 0 | 0 |  | 0 | 0 |  | 0 | 0 |  | 0 | 0 |  | 0 | 0 |  | 0 | 0 |
| Mongolia |  | 0 | 0 |  | 0 | 0 |  | 0 | 0 |  | 0 | 0 |  | 0 | 0 |  | 0 | 0 |
| Mauritania |  | 0 | 0 |  | 0 | 0 |  | 0 | 0 |  | 0 | 0 |  | 0 | 0 |  | 0 | 0 |
| Jamaica |  | 0 | 0 |  | 0 | 0 |  | 0 | 0 |  | 0 | 0 |  | 0 | 0 |  | 0 | 0 |
| Belize |  | 0 | 0 |  | 0 | 0 |  | 0 | 0 |  | 0 | 0 |  | 0 | 0 |  | 0 | 0 |
| Namibia |  | 0 | 0 |  | 0 | 0 |  | 0 | 0 |  | 0 | 0 |  | 0 | 0 |  | 0 | 0 |
| Somalia |  | 0 | 0 |  | 0.02 | 0.02 |  | 0 | 0 |  | 0 | 0 |  | 0 | 0 |  | 0 | 0 |
| Gaza Strip |  | 0 | 0 |  | 0 | 0 |  | 0 | 0 |  | 0 | 0 |  | 0 | 0 |  | 0 | 0 |
| Liechtenstein |  | 0 | 0 |  | 0 | 0 |  | 0 | 0 |  | 0 | 0 |  | 0 | 0 |  | 0 | 0 |
| Suriname |  | 0 | 0 |  | 0 | 0 |  | 0 | 0 |  | 0 | 0 |  | 0 | 0 |  | 0 | 0 |
| Andorra |  | 0 | 0 |  | 0 | 0 |  | 0 | 0 |  | 0 | 0 |  | 0 | 0 |  | 0 | 0 |
| Saudi Arabia |  | 0 | 0 |  | 0 | 0 |  | 0 | 0 |  | 0 | 0 |  | 0 | 0 |  | 0 | 0 |
| San Marino |  | 0 | 0 |  | 0 | 0 |  | 0 | 0 |  | 0 | 0 |  | 0 | 0 |  | 0 | 0 |
| Kuwait |  | 0 | 0 |  | 0 | 0 |  | 0 | 0 |  | 0 | 0 |  | 0 | 0 |  | 0 | 0 |
| Gibraltar |  | 0 | 0 |  | 0 | 0 |  | 0 | 0 |  | 0 | 0 |  | 0 | 0 |  | 0 | 0 |
| Isle of Man |  | 0 | 0 |  | 0 | 0 |  | 0 | 0 |  | 0 | 0 |  | 0 | 0 |  | 0 | 0 |
| Djibouti |  | 0 | 0 |  | 0 | 0 |  | 0 | 0 |  | 0 | 0 |  | 0 | 0 |  | 0 | 0 |
| Monaco |  | 0 | 0 |  | 0 | 0 |  | 0 | 0 |  | 0 | 0 |  | 0 | 0 |  | 0 | 0 |
| Vatican City |  | 0 | 0 |  | 0 | 0 |  | 0 | 0 |  | 0 | 0 |  | 0 | 0 |  | 0 | 0 |
