# Supplementary material for: Transformative optimisation of agricultural land use to meet future food demands
Source: PeerJ. 2013 Oct 24;1:e188. doi: 10.7717/peerj.188 (PMC3817586; doi:10.7717/peerj.188)
Supplement: Table S3 [file peerj-01-188-s005.docx]

**Table S3. Annual production of cereal crops in 171 countries under current and optimal land-use allocation options (all values in 10^6^ tons/y).**

|  |  | Barley | |  | Maize | |  | Millet | |  | Rice | |  | Sorghum | |  | Wheat | |
| --- | --- | --- | --- | --- | --- | --- | --- | --- | --- | --- | --- | --- | --- | --- | --- | --- | --- | --- |
| Country |  | Current | Optimal |  | Current | Optimal |  | Current | Optimal |  | Current | Optimal |  | Current | Optimal |  | Current | Optimal |
| China |  | 3.52 | 0.88 |  | 110.63 | 48.48 |  | 2.04 | 0 |  | 179 | 436.04 |  | 3.36 | 3.55 |  | 97.52 | 23.12 |
| United States |  | 6.07 | 5.67 |  | 242.52 | 432.08 |  | 0.24 | 0 |  | 9.66 | 2.75 |  | 11.98 | 3.54 |  | 59.45 | 5.28 |
| India |  | 1.48 | 23.85 |  | 9.26 | 24.93 |  | 9.65 | 0.1 |  | 128.14 | 187.41 |  | 7.66 | 0.41 |  | 72.4 | 68 |
| Russia |  | 17.62 | 0.66 |  | 2.17 | 60.08 |  | 0.8 | 0.03 |  | 0.72 | 17.11 |  | 0 | 1.25 |  | 39.76 | 12.58 |
| France |  | 10 | 0.01 |  | 13.87 | 72.12 |  | 0 | 0 |  | 0.19 | 0 |  | 0.46 | 0 |  | 35.77 | 0.49 |
| Indonesia |  | 0 | 0 |  | 8.14 | 0.52 |  | 0 | 0 |  | 48.92 | 61.23 |  | 0 | 0 |  | 0 | 0 |
| Australia |  | 10.22 | 14.58 |  | 0.81 | 58.9 |  | 0.1 | 0.2 |  | 2.58 | 45.25 |  | 2.92 | 0.88 |  | 31.97 | 1.27 |
| Canada |  | 12.5 | 4.33 |  | 8.63 | 98.74 |  | 0 | 0 |  | 0 | 0 |  | 0 | 0.01 |  | 25 | 0.32 |
| Brazil |  | 0.29 | 0.15 |  | 29.37 | 30.56 |  | 0 | 0 |  | 7.5 | 14.31 |  | 0.78 | 3.33 |  | 2.62 | 1.27 |
| Bangladesh |  | 0.01 | 0 |  | 0.03 | 1.34 |  | 0.04 | 0 |  | 35.73 | 37.2 |  | 0 | 0 |  | 1.83 | 0.47 |
| Argentina |  | 0.53 | 0.01 |  | 15.14 | 51.22 |  | 0.07 | 0 |  | 0.96 | 3.97 |  | 2.84 | 1.49 |  | 13.63 | 0.01 |
| Pakistan |  | 0.14 | 0.01 |  | 1.79 | 0.05 |  | 0.25 | 0 |  | 7.28 | 16.84 |  | 0.25 | 0 |  | 20.08 | 21.21 |
| Germany |  | 9.8 | 0.04 |  | 2.52 | 26.88 |  | 0 | 0 |  | 0 | 0 |  | 0 | 0 |  | 17.32 | 9.77 |
| Vietnam |  | 0 | 0.03 |  | 1.46 | 4.59 |  | 0 | 0 |  | 27.15 | 25.82 |  | 0 | 0 |  | 0.01 | 0 |
| Egypt |  | 0.2 | 0.1 |  | 10.15 | 9.47 |  | 0 | 0 |  | 7.05 | 22.68 |  | 1.77 | 2.35 |  | 9.12 | 1.29 |
| Turkey |  | 7.28 | 3.79 |  | 2.02 | 28.21 |  | 0.01 | 0.9 |  | 0.34 | 23.33 |  | 0 | 0 |  | 18.42 | 0.31 |
| Thailand |  | 0 | 1.15 |  | 3.54 | 29.47 |  | 0 | 0 |  | 24.27 | 5.9 |  | 0.15 | 0 |  | 0 | 0 |
| Ukraine |  | 8.32 | 0.02 |  | 4.16 | 10.99 |  | 0.29 | 0 |  | 0.07 | 26.15 |  | 0 | 0 |  | 14.5 | 4.01 |
| Mexico |  | 0.48 | 1.87 |  | 14.92 | 5.92 |  | 0 | 0 |  | 0.31 | 12.74 |  | 5.88 | 16.28 |  | 2.31 | 4.51 |
| Iran |  | 3.63 | 0.27 |  | 1.62 | 67.44 |  | 0.04 | 0.02 |  | 2.93 | 1.65 |  | 0 | 0 |  | 14.27 | 0.5 |
| Others (below) |  | 57.78 | 27.1 |  | 98.3 | 265.31 |  | 12.12 | 12.72 |  | 85.05 | 187.05 |  | 18.68 | 45.23 |  | 135.28 | 131.14 |
| United Kingdom |  | 7.32 | 0.04 |  | 0 | 0 |  | 0 | 0 |  | 0 | 0 |  | 0 | 0 |  | 13.82 | 23.91 |
| Myanmar |  | 0 | 0.03 |  | 0.45 | 0.19 |  | 0.17 | 0 |  | 19.46 | 21.1 |  | 0 | 0 |  | 0.15 | 0.01 |
| Spain |  | 8.94 | 0.01 |  | 4.31 | 58.84 |  | 0 | 0 |  | 0.95 | 0.51 |  | 0 | 0.46 |  | 5.97 | 0.08 |
| Italy |  | 0.94 | 0 |  | 10.94 | 27.46 |  | 0 | 0 |  | 1.4 | 0.12 |  | 0.24 | 1.03 |  | 5.34 | 0 |
| Nigeria |  | 0 | 0 |  | 4.47 | 1.51 |  | 4.92 | 0.11 |  | 3.04 | 4.45 |  | 6.33 | 0.1 |  | 0.07 | 20.75 |
| Romania |  | 1.09 | 0.98 |  | 9.04 | 14.85 |  | 0 | 0.01 |  | 0.01 | 0.35 |  | 0 | 0.05 |  | 5.14 | 0.9 |
| Philippines |  | 0 | 0 |  | 4.02 | 1.32 |  | 0 | 0 |  | 11.02 | 17.15 |  | 0 | 0 |  | 0 | 0 |
| Kazakhstan |  | 2 | 0.18 |  | 0.34 | 3.61 |  | 0.07 | 0 |  | 0.27 | 0.27 |  | 0 | 32.18 |  | 11.27 | 0.09 |
| Poland |  | 3.45 | 0.05 |  | 0.94 | 20.82 |  | 0 | 0 |  | 0 | 0.01 |  | 0 | 0 |  | 9.12 | 0.04 |
| Hungary |  | 1.09 | 0 |  | 6.42 | 13.95 |  | 0.01 | 0 |  | 0.01 | 0.01 |  | 0.02 | 0 |  | 4.1 | 0.14 |
| Syria |  | 2.22 | 0.05 |  | 0.45 | 19.14 |  | 0 | 0.09 |  | 0 | 1.83 |  | 0.01 | 0.03 |  | 8.79 | 7.49 |
| Japan |  | 0.22 | 0.03 |  | 0 | 0 |  | 0 | 0 |  | 9.54 | 11.02 |  | 0 | 0 |  | 0.58 | 0 |
| South Africa |  | 0.24 | 0.08 |  | 7.6 | 5.44 |  | 0.02 | 0 |  | 0 | 1.04 |  | 0.3 | 3.78 |  | 1.83 | 5.38 |
| Saudi Arabia |  | 0.82 | 9.23 |  | 0.04 | 0.22 |  | 0.04 | 0.15 |  | 0 | 0 |  | 0.46 | 0.24 |  | 8.41 | 2.03 |
| Denmark |  | 4.39 | 0.08 |  | 0 | 1.53 |  | 0 | 0 |  | 0 | 0 |  | 0 | 0 |  | 4.96 | 9.45 |
| Morocco |  | 2.45 | 0.09 |  | 0.27 | 2.81 |  | 0.02 | 4.59 |  | 0.04 | 11.97 |  | 0.02 | 0 |  | 4.65 | 1.25 |
| Serbia |  | 0.35 | 0 |  | 4.9 | 3.57 |  | 0 | 5.66 |  | 0 | 0.09 |  | 0 | 0.13 |  | 2.12 | 0.08 |
| South Korea |  | 0.25 | 0 |  | 0.08 | 0 |  | 0 | 0 |  | 6 | 6.63 |  | 0 | 0 |  | 0.02 | 0 |
| Uzbekistan |  | 0.12 | 0 |  | 0.15 | 7.27 |  | 0 | 0 |  | 0.27 | 0.09 |  | 0.03 | 0.09 |  | 5.56 | 0.25 |
| Nepal |  | 0.03 | 0.13 |  | 1.19 | 0.07 |  | 0.21 | 0 |  | 3.47 | 7 |  | 0 | 0 |  | 1.16 | 0.28 |
| Bulgaria |  | 0.84 | 1.51 |  | 1.42 | 3.1 |  | 0.01 | 0.02 |  | 0.06 | 1.58 |  | 0.02 | 0 |  | 3.24 | 0.64 |
| Sweden |  | 2.58 | 0.14 |  | 0 | 0 |  | 0 | 0 |  | 0 | 0 |  | 0 | 0 |  | 2.91 | 6.53 |
| Czech Republic |  | 1.56 | 0 |  | 0.33 | 7.93 |  | 0.01 | 0 |  | 0 | 0 |  | 0 | 0 |  | 3.32 | 0.12 |
| Ethiopia |  | 0.73 | 0.18 |  | 2.24 | 4.72 |  | 0.22 | 0.17 |  | 0 | 0.06 |  | 1.12 | 1.44 |  | 0.84 | 0.71 |
| Austria |  | 1.24 | 0 |  | 2.37 | 7.62 |  | 0.01 | 0 |  | 0 | 0 |  | 0 | 0.01 |  | 1.52 | 0.03 |
| Algeria |  | 1.2 | 1.91 |  | 0.01 | 2.18 |  | 0 | 0.06 |  | 0 | 5.46 |  | 0 | 0.04 |  | 3.83 | 0.97 |
| Cambodia |  | 0 | 0.28 |  | 0.14 | 1.63 |  | 0 | 0 |  | 4.15 | 3.13 |  | 0 | 0.03 |  | 0 | 0 |
| Sudan |  | 0 | 0 |  | 0.1 | 0.06 |  | 0.59 | 0.01 |  | 0 | 5.89 |  | 2.81 | 0.01 |  | 0.55 | 6.25 |
| Finland |  | 3.16 | 0.94 |  | 0 | 0.02 |  | 0 | 0 |  | 0 | 0 |  | 0 | 0 |  | 0.84 | 3.57 |
| Greece |  | 0.24 | 0 |  | 1.58 | 8.83 |  | 0 | 0 |  | 0.16 | 0.62 |  | 0 | 0.01 |  | 1.69 | 0.01 |
| North Korea |  | 0.08 | 0.03 |  | 1.27 | 0.21 |  | 0.04 | 0 |  | 1.83 | 3.82 |  | 0.02 | 0.07 |  | 0.12 | 0 |
| Croatia |  | 0.14 | 0 |  | 2.25 | 3.74 |  | 0 | 0 |  | 0 | 0 |  | 0 | 0.03 |  | 0.92 | 0.02 |
| Colombia |  | 0.02 | 0.02 |  | 0.89 | 0.27 |  | 0 | 0 |  | 2.02 | 3.32 |  | 0.26 | 0.99 |  | 0.05 | 0.1 |
| Tanzania |  | 0 | 3.76 |  | 2.01 | 0.7 |  | 0.21 | 0 |  | 0.42 | 0.55 |  | 0.48 | 0 |  | 0.08 | 0 |
| Slovakia |  | 0.57 | 0.02 |  | 0.78 | 3.12 |  | 0 | 0 |  | 0 | 0 |  | 0 | 0 |  | 1.52 | 0.35 |
| Kenya |  | 0.11 | 1.62 |  | 2.16 | 0.03 |  | 0.07 | 0 |  | 0.06 | 4.41 |  | 0.11 | 0 |  | 0.25 | 0.01 |
| Niger |  | 0 | 0 |  | 0.09 | 0.24 |  | 1.95 | 0 |  | 0.11 | 4.23 |  | 0.51 | 0 |  | 0.01 | 9.81 |
| New Zealand |  | 1.07 | 0.03 |  | 0.5 | 3.95 |  | 0 | 0 |  | 0 | 0 |  | 0 | 0 |  | 0.91 | 0.3 |
| Peru |  | 0.13 | 0 |  | 0.73 | 0.01 |  | 0 | 0 |  | 1.4 | 4.39 |  | 0 | 0.72 |  | 0.17 | 0.01 |
| Belarus |  | 1.54 | 0 |  | 0.06 | 2.71 |  | 0 | 0 |  | 0 | 0.16 |  | 0 | 0 |  | 0.83 | 0.51 |
| Afghanistan |  | 0.12 | 0.11 |  | 0.14 | 0.23 |  | 0.04 | 0 |  | 0.21 | 3.47 |  | 0 | 0.31 |  | 1.88 | 0.1 |
| Sri Lanka |  | 0 | 0 |  | 0.03 | 0.03 |  | 0.01 | 0 |  | 2.32 | 2.4 |  | 0 | 0 |  | 0 | 0 |
| Uganda |  | 0 | 0.22 |  | 1.15 | 0.61 |  | 0.6 | 0.27 |  | 0.15 | 1.78 |  | 0.41 | 0.01 |  | 0.03 | 0.13 |
| Moldova |  | 0.2 | 0.16 |  | 1.24 | 1.18 |  | 0 | 0 |  | 0 | 0.71 |  | 0 | 0 |  | 0.84 | 1.03 |
| Malaysia |  | 0 | 0 |  | 0.07 | 0.5 |  | 0 | 0 |  | 2.18 | 1.9 |  | 0 | 0 |  | 0 | 0 |
| Venezuela |  | 0 | 0 |  | 1.02 | 0.07 |  | 0 | 0 |  | 0.67 | 3.4 |  | 0.46 | 0.01 |  | 0 | 0 |
| Lithuania |  | 0.88 | 0.04 |  | 0 | 0.43 |  | 0 | 0 |  | 0 | 0 |  | 0 | 0 |  | 1.04 | 1.83 |
| Chile |  | 0.07 | 0.01 |  | 0.7 | 2.62 |  | 0 | 0 |  | 0.14 | 0.01 |  | 0 | 0 |  | 1.02 | 0.26 |
| Laos |  | 0 | 0.09 |  | 0.11 | 0.3 |  | 0 | 0 |  | 1.76 | 1.61 |  | 0 | 0 |  | 0 | 0 |
| Turkmenistan |  | 0.04 | 0.01 |  | 0.02 | 0.29 |  | 0 | 0 |  | 0.07 | 0.06 |  | 0 | 0 |  | 1.73 | 1.91 |
| Tunisia |  | 0.3 | 0.06 |  | 0 | 0 |  | 0 | 0 |  | 0 | 0.19 |  | 0 | 0.18 |  | 1.56 | 1.83 |
| Burkina Faso |  | 0 | 0 |  | 0.24 | 0.57 |  | 0.65 | 0 |  | 0.09 | 3.73 |  | 0.87 | 0 |  | 0 | 0.01 |
| Congo, DRC |  | 0 | 0.01 |  | 1.23 | 0.07 |  | 0.06 | 0.09 |  | 0.44 | 0.11 |  | 0.1 | 0.04 |  | 0 | 2.76 |
| Madagascar |  | 0 | 0 |  | 0.11 | 0 |  | 0 | 0 |  | 1.71 | 0.85 |  | 0 | 0 |  | 0.01 | 1.56 |
| Cote d'Ivoire |  | 0 | 0 |  | 0.64 | 0.01 |  | 0.05 | 0 |  | 1.11 | 3.08 |  | 0.03 | 0 |  | 0 | 0 |
| Uruguay |  | 0.22 | 0 |  | 0.19 | 0.03 |  | 0 | 0 |  | 0.88 | 3.23 |  | 0.09 | 0.09 |  | 0.33 | 0 |
| Mali |  | 0 | 0.03 |  | 0.17 | 0.5 |  | 0.61 | 0.01 |  | 0.5 | 2.76 |  | 0.36 | 0.03 |  | 0.01 | 0.02 |
| Ireland |  | 1.1 | 0 |  | 0 | 0 |  | 0 | 0 |  | 0 | 0 |  | 0 | 0 |  | 0.49 | 1.97 |
| Kyrgyzstan |  | 0.15 | 0 |  | 0.3 | 3.08 |  | 0 | 0 |  | 0.05 | 0.04 |  | 0 | 0.01 |  | 1.04 | 0 |
| Azerbaijan |  | 0.21 | 0 |  | 0.09 | 0.21 |  | 0 | 0 |  | 0.05 | 3.15 |  | 0 | 0 |  | 1.13 | 0.01 |
| Yemen |  | 0.13 | 0.09 |  | 0.1 | 1.66 |  | 0.11 | 0.01 |  | 0 | 0 |  | 0.64 | 0.02 |  | 0.46 | 1.49 |
| Switzerland |  | 0.41 | 0 |  | 0.23 | 2.12 |  | 0 | 0 |  | 0.01 | 0 |  | 0 | 0 |  | 0.8 | 0 |
| Guatemala |  | 0 | 0 |  | 1.3 | 0.09 |  | 0 | 0 |  | 0.05 | 1.84 |  | 0.05 | 0.07 |  | 0.02 | 0.16 |
| Ecuador |  | 0.03 | 0 |  | 0.47 | 0.08 |  | 0 | 0 |  | 0.87 | 2.03 |  | 0.01 | 0.09 |  | 0.02 | 0.01 |
| Ghana |  | 0 | 0 |  | 0.87 | 0.27 |  | 0.1 | 0 |  | 0.19 | 1.71 |  | 0.22 | 0 |  | 0 | 0 |
| Cameroon |  | 0 | 0 |  | 0.7 | 0.14 |  | 0.07 | 0.01 |  | 0.15 | 2.78 |  | 0.47 | 0.02 |  | 0 | 0.03 |
| Norway |  | 0.89 | 0.01 |  | 0 | 0 |  | 0 | 0 |  | 0 | 0 |  | 0 | 0 |  | 0.48 | 1.61 |
| Zimbabwe |  | 0.08 | 3.7 |  | 0.89 | 0 |  | 0.04 | 0 |  | 0 | 0 |  | 0.06 | 0 |  | 0.23 | 2.57 |
| Bolivia |  | 0.09 | 0.02 |  | 0.56 | 1.44 |  | 0 | 0 |  | 0.34 | 0.27 |  | 0.15 | 0.24 |  | 0.14 | 0 |
| Mozambique |  | 0 | 0.35 |  | 0.83 | 0.46 |  | 0.06 | 0.03 |  | 0.13 | 0.89 |  | 0.2 | 0.14 |  | 0.01 | 0.46 |
| Portugal |  | 0.07 | 0 |  | 0.72 | 2.12 |  | 0 | 0 |  | 0.11 | 0.5 |  | 0 | 0.08 |  | 0.24 | 0 |
| Malawi |  | 0 | 0.03 |  | 1.02 | 0.39 |  | 0.01 | 0 |  | 0.05 | 0.91 |  | 0.02 | 0 |  | 0.01 | 0.24 |
| Netherlands |  | 0.19 | 0.02 |  | 0.13 | 0.42 |  | 0 | 0 |  | 0 | 0 |  | 0 | 0 |  | 0.73 | 0.77 |
| Paraguay |  | 0 | 0 |  | 0.64 | 0.23 |  | 0 | 0 |  | 0.13 | 1.77 |  | 0.04 | 0.01 |  | 0.23 | 0 |
| Bosnia & Herzegovina |  | 0.06 | 0.04 |  | 0.72 | 0.89 |  | 0 | 0.2 |  | 0 | 0 |  | 0 | 0.07 |  | 0.24 | 0.04 |
| Cuba |  | 0 | 0 |  | 0.31 | 0 |  | 0 | 0 |  | 0.66 | 1.11 |  | 0 | 0 |  | 0 | 0 |
| Zambia |  | 0 | 0.02 |  | 0.63 | 0.06 |  | 0.06 | 0 |  | 0.03 | 0.01 |  | 0.04 | 0 |  | 0.12 | 3.1 |
| Nicaragua |  | 0 | 0 |  | 0.35 | 0.01 |  | 0 | 0 |  | 0.27 | 1.14 |  | 0.1 | 0.2 |  | 0 | 0 |
| Senegal |  | 0 | 0 |  | 0.07 | 0.33 |  | 0.34 | 0.01 |  | 0.21 | 1.03 |  | 0.09 | 0.01 |  | 0 | 0 |
| Belgium |  | 0.12 | 0.05 |  | 0.14 | 0.65 |  | 0 | 0 |  | 0 | 0 |  | 0 | 0.01 |  | 0.45 | 0.18 |
| Guinea |  | 0 | 0 |  | 0.07 | 0 |  | 0.02 | 0 |  | 0.58 | 0.75 |  | 0.02 | 0 |  | 0 | 0 |
| Iraq |  | 0.12 | 0 |  | 0.17 | 1.29 |  | 0 | 0 |  | 0.1 | 0.25 |  | 0 | 0 |  | 0.27 | 0.07 |
| Latvia |  | 0.27 | 0.02 |  | 0 | 0.08 |  | 0 | 0 |  | 0 | 0 |  | 0 | 0 |  | 0.4 | 0.69 |
| El Salvador |  | 0 | 0 |  | 0.49 | 0.01 |  | 0 | 0 |  | 0.03 | 2.09 |  | 0.13 | 0 |  | 0 | 0 |
| Chad |  | 0 | 0 |  | 0.11 | 0.02 |  | 0.27 | 0 |  | 0.04 | 0.75 |  | 0.23 | 0 |  | 0 | 1.63 |
| Macedonia |  | 0.1 | 0 |  | 0.15 | 0.83 |  | 0 | 0 |  | 0.02 | 0.61 |  | 0 | 0 |  | 0.37 | 0 |
| Benin |  | 0 | 0 |  | 0.39 | 0.12 |  | 0.05 | 0 |  | 0.06 | 0.91 |  | 0.13 | 0.01 |  | 0 | 0.04 |
| Slovenia |  | 0.05 | 0 |  | 0.5 | 0.72 |  | 0 | 0 |  | 0 | 0 |  | 0 | 0 |  | 0.06 | 0 |
| Togo |  | 0 | 0 |  | 0.36 | 0.01 |  | 0.03 | 0 |  | 0.06 | 1.32 |  | 0.15 | 0 |  | 0 | 0 |
| Dominican Republic |  | 0 | 0 |  | 0.03 | 0 |  | 0 | 0 |  | 0.55 | 0.68 |  | 0.01 | 0 |  | 0 | 0 |
| Montenegro |  | 0.02 | 0 |  | 0.36 | 0 |  | 0 | 0.98 |  | 0 | 0 |  | 0 | 0 |  | 0.2 | 0 |
| Angola |  | 0 | 0 |  | 0.41 | 0 |  | 0.12 | 0 |  | 0.01 | 1.42 |  | 0 | 0 |  | 0 | 0.37 |
| Honduras |  | 0 | 0 |  | 0.41 | 0.03 |  | 0 | 0 |  | 0.02 | 0.96 |  | 0.06 | 0.08 |  | 0 | 0.01 |
| Tajikistan |  | 0.03 | 0 |  | 0.06 | 0.15 |  | 0 | 0 |  | 0.05 | 0.03 |  | 0 | 1.25 |  | 0.36 | 0 |
| Georgia |  | 0.04 | 0 |  | 0.31 | 0.45 |  | 0 | 0 |  | 0 | 0.12 |  | 0 | 0 |  | 0.14 | 0.03 |
| Libya |  | 0.14 | 0.01 |  | 0.01 | 1.84 |  | 0.02 | 0.07 |  | 0 | 0 |  | 0 | 0 |  | 0.33 | 0.09 |
| Albania |  | 0.01 | 0 |  | 0.15 | 0.34 |  | 0 | 0.1 |  | 0 | 0.01 |  | 0 | 0 |  | 0.27 | 0.12 |
| Haiti |  | 0 | 0 |  | 0.21 | 0 |  | 0 | 0 |  | 0.13 | 0.92 |  | 0.08 | 0 |  | 0 | 0 |
| Somalia |  | 0 | 0 |  | 0.23 | 0.01 |  | 0 | 0 |  | 0 | 1.51 |  | 0.17 | 0 |  | 0.01 | 0 |
| Guyana |  | 0 | 0 |  | 0.01 | 0.02 |  | 0 | 0 |  | 0.38 | 0.41 |  | 0 | 0 |  | 0 | 0 |
| Suriname |  | 0 | 0 |  | 0 | 0 |  | 0 | 0 |  | 0.35 | 0.35 |  | 0 | 0 |  | 0 | 0 |
| Estonia |  | 0.2 | 0.15 |  | 0 | 0.03 |  | 0 | 0 |  | 0 | 0 |  | 0 | 0 |  | 0.11 | 0.17 |
| Liberia |  | 0 | 0 |  | 0 | 0.01 |  | 0 | 0 |  | 0.3 | 0.31 |  | 0 | 0 |  | 0 | 0 |
| Sierra Leone |  | 0 | 0 |  | 0.01 | 0.04 |  | 0.01 | 0.03 |  | 0.26 | 0.25 |  | 0.01 | 0 |  | 0 | 0 |
| Lebanon |  | 0.06 | 0 |  | 0.01 | 0.37 |  | 0 | 0 |  | 0 | 0 |  | 0 | 0 |  | 0.22 | 0.18 |
| Armenia |  | 0.07 | 0 |  | 0.01 | 0.42 |  | 0 | 0 |  | 0 | 0.22 |  | 0 | 0 |  | 0.2 | 0 |
| Burundi |  | 0 | 0.04 |  | 0.14 | 0.03 |  | 0.01 | 0.01 |  | 0.04 | 0.39 |  | 0.05 | 0.02 |  | 0.01 | 0 |
| Costa Rica |  | 0 | 0 |  | 0.02 | 0 |  | 0 | 0 |  | 0.22 | 0.28 |  | 0 | 0.01 |  | 0 | 0 |
| Panama |  | 0 | 0 |  | 0.06 | 0.04 |  | 0 | 0 |  | 0.17 | 0.15 |  | 0.01 | 0.16 |  | 0 | 0 |
| Central African Republic |  | 0 | 0 |  | 0.11 | 0.01 |  | 0.02 | 0 |  | 0.04 | 0.34 |  | 0.05 | 0 |  | 0 | 0.03 |
| Mongolia |  | 0.01 | 0.12 |  | 0.02 | 0.13 |  | 0 | 0 |  | 0 | 0.03 |  | 0 | 0.01 |  | 0.17 | 0.03 |
| Rwanda |  | 0 | 0.03 |  | 0.05 | 0.01 |  | 0 | 0 |  | 0.01 | 0.53 |  | 0.11 | 0.01 |  | 0.01 | 0.02 |
| Israel |  | 0.03 | 0 |  | 0.06 | 0.78 |  | 0 | 0 |  | 0 | 0 |  | 0.01 | 0.18 |  | 0.08 | 0 |
| The Gambia |  | 0 | 0 |  | 0.01 | 0.04 |  | 0.11 | 0 |  | 0.04 | 0.25 |  | 0.01 | 0 |  | 0 | 0 |
| Eritrea |  | 0.02 | 0.01 |  | 0.01 | 0.05 |  | 0.01 | 0 |  | 0 | 0.09 |  | 0.1 | 0.09 |  | 0.01 | 0.04 |
| Lesotho |  | 0 | 0 |  | 0.12 | 0.02 |  | 0 | 0 |  | 0 | 0.05 |  | 0.02 | 0.1 |  | 0.02 | 0.11 |
| Jordan |  | 0.06 | 0 |  | 0.07 | 1.09 |  | 0 | 0 |  | 0 | 0 |  | 0 | 0 |  | 0.03 | 0 |
| Timor-Leste |  | 0 | 0 |  | 0.1 | 0.01 |  | 0 | 0 |  | 0.05 | 0.22 |  | 0 | 0 |  | 0 | 0 |
| Mauritania |  | 0 | 0.14 |  | 0.01 | 0 |  | 0 | 0 |  | 0.07 | 0.42 |  | 0.06 | 0 |  | 0 | 0 |
| Bhutan |  | 0 | 0 |  | 0.04 | 0.05 |  | 0.01 | 0 |  | 0.08 | 0.09 |  | 0 | 0 |  | 0.01 | 0.04 |
| Guinea-Bissau |  | 0 | 0 |  | 0.02 | 0 |  | 0.02 | 0 |  | 0.08 | 0.15 |  | 0.01 | 0 |  | 0 | 0 |
| Swaziland |  | 0 | 0 |  | 0.1 | 0.02 |  | 0 | 0 |  | 0 | 0.27 |  | 0 | 0 |  | 0 | 0 |
| Namibia |  | 0 | 0 |  | 0.06 | 0 |  | 0.03 | 0 |  | 0 | 0 |  | 0 | 0 |  | 0 | 1.05 |
| Botswana |  | 0 | 0.08 |  | 0.02 | 0.01 |  | 0 | 0 |  | 0 | 0.04 |  | 0.02 | 0.01 |  | 0.01 | 0.18 |
| West Bank |  | 0 | 0 |  | 0.02 | 0.14 |  | 0 | 0 |  | 0 | 0 |  | 0 | 0.12 |  | 0.02 | 0 |
| Gabon |  | 0 | 0 |  | 0.04 | 0.01 |  | 0 | 0 |  | 0 | 0.05 |  | 0 | 0 |  | 0 | 0 |
| Belize |  | 0 | 0 |  | 0.02 | 0.01 |  | 0 | 0 |  | 0.01 | 0.01 |  | 0.01 | 0.03 |  | 0 | 0 |
| Luxembourg |  | 0.02 | 0 |  | 0 | 0.02 |  | 0 | 0 |  | 0 | 0 |  | 0 | 0 |  | 0.02 | 0.02 |
| Oman |  | 0 | 0 |  | 0 | 0 |  | 0 | 0 |  | 0 | 0 |  | 0.02 | 0.01 |  | 0.01 | 0.03 |
| French Guiana |  | 0 | 0 |  | 0 | 0 |  | 0 | 0 |  | 0.03 | 0.03 |  | 0 | 0 |  | 0 | 0 |
| Kuwait |  | 0.03 | 0 |  | 0 | 0.25 |  | 0 | 0 |  | 0 | 0 |  | 0 | 0 |  | 0 | 0 |
| Congo |  | 0 | 0 |  | 0.02 | 0.02 |  | 0 | 0 |  | 0 | 0.01 |  | 0 | 0 |  | 0 | 0.01 |
| Trinidad & Tobago |  | 0 | 0 |  | 0 | 0 |  | 0 | 0 |  | 0.01 | 0.01 |  | 0 | 0 |  | 0 | 0 |
| United Arab Emirates |  | 0 | 0 |  | 0 | 0 |  | 0 | 0 |  | 0 | 0 |  | 0.01 | 0 |  | 0 | 0.01 |
| Liechtenstein |  | 0 | 0 |  | 0 | 0.01 |  | 0 | 0 |  | 0 | 0 |  | 0 | 0 |  | 0 | 0 |
| Equatorial Guinea |  | 0 | 0 |  | 0 | 0 |  | 0 | 0 |  | 0 | 0.02 |  | 0 | 0 |  | 0 | 0 |
| Papua New Guinea |  | 0 | 0 |  | 0 | 0 |  | 0 | 0 |  | 0 | 0 |  | 0 | 0 |  | 0 | 0 |
| San Marino |  | 0 | 0 |  | 0 | 0 |  | 0 | 0 |  | 0 | 0 |  | 0 | 0 |  | 0 | 0 |
| Jamaica |  | 0 | 0 |  | 0 | 0 |  | 0 | 0 |  | 0 | 0 |  | 0 | 0 |  | 0 | 0 |
| Andorra |  | 0 | 0 |  | 0 | 0 |  | 0 | 0 |  | 0 | 0 |  | 0 | 0 |  | 0 | 0 |
| Gaza Strip |  | 0 | 0 |  | 0 | 0.01 |  | 0 | 0 |  | 0 | 0 |  | 0 | 0 |  | 0 | 0 |
| Puerto Rico |  | 0 | 0 |  | 0 | 0 |  | 0 | 0 |  | 0 | 0 |  | 0 | 0 |  | 0 | 0 |
| Brunei |  | 0 | 0 |  | 0 | 0 |  | 0 | 0 |  | 0 | 0 |  | 0 | 0 |  | 0 | 0 |
| Bahrain |  | 0 | 0 |  | 0 | 0 |  | 0 | 0 |  | 0 | 0 |  | 0 | 0 |  | 0 | 0 |
| Singapore |  | 0 | 0 |  | 0 | 0 |  | 0 | 0 |  | 0 | 0 |  | 0 | 0 |  | 0 | 0 |
| Isle of Man |  | 0 | 0 |  | 0 | 0 |  | 0 | 0 |  | 0 | 0 |  | 0 | 0 |  | 0 | 0 |
| Vatican City |  | 0 | 0 |  | 0 | 0 |  | 0 | 0 |  | 0 | 0 |  | 0 | 0 |  | 0 | 0 |
| Monaco |  | 0 | 0 |  | 0 | 0 |  | 0 | 0 |  | 0 | 0 |  | 0 | 0 |  | 0 | 0 |
| Gibraltar |  | 0 | 0 |  | 0 | 0 |  | 0 | 0 |  | 0 | 0 |  | 0 | 0 |  | 0 | 0 |
| Jersey |  | 0 | 0 |  | 0 | 0 |  | 0 | 0 |  | 0 | 0 |  | 0 | 0 |  | 0 | 0 |
