# Supplementary material for: Transformative optimisation of agricultural land use to meet future food demands
Source: PeerJ. 2013 Oct 24;1:e188. doi: 10.7717/peerj.188 (PMC3817586; doi:10.7717/peerj.188)
Supplement: Table S5 [file peerj-01-188-s007.docx]

**Table S5. Potential annual revenue generated from cereal crops in 171 countries under current and optimal land-use allocation options (all values in 10^6^ US$/y). Calculations were based on producer prices of each crop averaged among all producer countries over the period 2000-2007 (Source for price data: FAOSTAT. Statistical Databases and Data-Sets, Rome, Italy, Food and Agriculture Organization of the United Nations, 2010).**

|  |  | Barley | |  | Maize | |  | Millet | |  | Rice | |  | Sorghum | |  | Wheat | |
| --- | --- | --- | --- | --- | --- | --- | --- | --- | --- | --- | --- | --- | --- | --- | --- | --- | --- | --- |
| Country |  | Current | Optimal |  | Current | Optimal |  | Current | Optimal |  | Current | Optimal |  | Current | Optimal |  | Current | Optimal |
| China |  | 654.1 | 164.4 |  | 28640.1 | 12550.1 |  | 517.4 | 0.8 |  | 64720.1 | 157657.4 |  | 715.5 | 756.5 |  | 20726.7 | 4913.4 |
| United States |  | 1129.8 | 1054.5 |  | 62783.4 | 111857.7 |  | 61.3 | 0.0 |  | 3494.3 | 995.0 |  | 2553.6 | 755.2 |  | 12634.9 | 1123.1 |
| India |  | 275.2 | 4437.3 |  | 2396.8 | 6454.4 |  | 2445.5 | 24.4 |  | 46329.6 | 67759.3 |  | 1632.7 | 87.8 |  | 15388.4 | 14453.0 |
| Australia |  | 1901.3 | 2713.0 |  | 208.4 | 15247.0 |  | 24.3 | 50.0 |  | 932.8 | 16362.2 |  | 622.2 | 186.6 |  | 6795.5 | 269.8 |
| Canada |  | 2326.8 | 806.0 |  | 2234.0 | 25562.5 |  | 0.1 | 0.0 |  | 0.0 | 0.0 |  | 0.0 | 1.7 |  | 5313.5 | 67.9 |
| Iran |  | 675.1 | 50.6 |  | 418.9 | 17458.9 |  | 10.7 | 5.8 |  | 1060.8 | 597.5 |  | 0.1 | 0.1 |  | 3032.5 | 106.9 |
| Russia |  | 3279.1 | 122.4 |  | 560.6 | 15554.1 |  | 202.7 | 7.5 |  | 260.1 | 6184.6 |  | 0.2 | 265.7 |  | 8450.6 | 2674.2 |
| Spain |  | 1663.4 | 1.3 |  | 1115.4 | 15231.7 |  | 0.0 | 0.0 |  | 343.6 | 184.2 |  | 0.1 | 98.2 |  | 1269.6 | 16.9 |
| Turkey |  | 1355.1 | 704.6 |  | 522.3 | 7302.3 |  | 2.8 | 228.6 |  | 122.8 | 8434.1 |  | 0.1 | 0.0 |  | 3914.2 | 66.3 |
| Ukraine |  | 1548.1 | 4.2 |  | 1076.9 | 2845.0 |  | 73.1 | 0.0 |  | 26.9 | 9454.9 |  | 0.1 | 0.0 |  | 3081.0 | 852.8 |
| Argentina |  | 98.7 | 1.8 |  | 3919.8 | 13259.7 |  | 18.0 | 0.6 |  | 347.1 | 1434.0 |  | 604.4 | 318.0 |  | 2897.4 | 2.1 |
| France |  | 1861.3 | 1.4 |  | 3591.0 | 18671.6 |  | 0.0 | 0.0 |  | 67.4 | 0.1 |  | 98.7 | 0.5 |  | 7601.9 | 103.7 |
| Mexico |  | 88.9 | 348.5 |  | 3862.7 | 1532.5 |  | 0.0 | 0.0 |  | 110.5 | 4605.2 |  | 1252.5 | 3470.7 |  | 491.5 | 957.5 |
| Kazakhstan |  | 372.6 | 33.1 |  | 88.2 | 933.8 |  | 18.1 | 0.6 |  | 97.6 | 96.9 |  | 0.5 | 6858.4 |  | 2394.7 | 19.8 |
| Morocco |  | 456.4 | 16.8 |  | 69.8 | 726.3 |  | 5.8 | 1163.1 |  | 14.7 | 4329.4 |  | 4.1 | 0.0 |  | 988.8 | 266.2 |
| Syria |  | 413.8 | 9.8 |  | 115.9 | 4953.7 |  | 0.0 | 23.3 |  | 0.1 | 661.6 |  | 1.8 | 7.3 |  | 1868.6 | 1591.2 |
| Egypt |  | 36.4 | 18.6 |  | 2628.8 | 2451.3 |  | 0.0 | 0.0 |  | 2550.1 | 8199.2 |  | 378.0 | 500.4 |  | 1937.8 | 274.5 |
| Pakistan |  | 25.5 | 1.4 |  | 463.7 | 12.9 |  | 62.2 | 0.7 |  | 2631.4 | 6089.3 |  | 52.8 | 0.4 |  | 4267.3 | 4507.9 |
| Niger |  | 0.0 | 0.0 |  | 24.1 | 62.6 |  | 494.1 | 0.5 |  | 41.2 | 1530.0 |  | 109.7 | 0.6 |  | 1.9 | 2084.7 |
| Brazil |  | 53.9 | 27.4 |  | 7603.3 | 7911.4 |  | 0.0 | 0.0 |  | 2712.5 | 5173.7 |  | 165.9 | 710.4 |  | 556.2 | 270.7 |
| Others (below) |  | 9671.0 | 5207.5 |  | 28098.6 | 63033.4 |  | 2563.9 | 2035.7 |  | 79456.5 | 107885.6 |  | 3897.0 | 2675.5 |  | 26300.0 | 26071.6 |
| Germany |  | 1824.1 | 6.6 |  | 653.3 | 6957.7 |  | 0.0 | 0.0 |  | 0.0 | 0.0 |  | 0.4 | 0.0 |  | 3680.6 | 2077.4 |
| Italy |  | 174.4 | 0.0 |  | 2831.4 | 7108.1 |  | 0.0 | 0.0 |  | 504.8 | 43.4 |  | 51.8 | 219.5 |  | 1134.0 | 0.0 |
| Poland |  | 642.3 | 8.7 |  | 242.2 | 5390.2 |  | 0.5 | 0.0 |  | 0.0 | 5.0 |  | 0.0 | 0.0 |  | 1937.9 | 9.1 |
| Sudan |  | 0.6 | 0.5 |  | 26.8 | 16.8 |  | 148.9 | 3.4 |  | 0.6 | 2128.9 |  | 598.5 | 3.1 |  | 117.8 | 1327.4 |
| Indonesia |  | 0.0 | 0.0 |  | 2107.6 | 135.4 |  | 0.0 | 0.0 |  | 17689.1 | 22137.5 |  | 0.0 | 0.8 |  | 0.0 | 0.0 |
| Algeria |  | 223.0 | 356.2 |  | 1.3 | 565.6 |  | 0.0 | 15.5 |  | 0.0 | 1973.9 |  | 0.1 | 7.5 |  | 814.0 | 207.2 |
| Greece |  | 43.9 | 0.0 |  | 410.1 | 2285.7 |  | 0.0 | 0.0 |  | 59.5 | 225.4 |  | 0.0 | 1.7 |  | 359.8 | 2.2 |
| Nigeria |  | 0.0 | 0.0 |  | 1156.9 | 391.4 |  | 1246.8 | 26.6 |  | 1099.0 | 1608.0 |  | 1348.5 | 20.5 |  | 14.9 | 4409.4 |
| Philippines |  | 0.0 | 0.0 |  | 1039.9 | 341.2 |  | 0.0 | 0.0 |  | 3985.7 | 6200.0 |  | 0.0 | 0.0 |  | 0.0 | 0.0 |
| South Africa |  | 43.9 | 15.5 |  | 1966.6 | 1409.4 |  | 4.7 | 0.0 |  | 0.0 | 375.0 |  | 63.5 | 806.4 |  | 389.4 | 1142.8 |
| Kenya |  | 20.8 | 301.8 |  | 558.7 | 6.6 |  | 17.0 | 0.4 |  | 20.1 | 1594.9 |  | 22.6 | 0.0 |  | 52.3 | 2.6 |
| Burkina Faso |  | 0.0 | 0.0 |  | 62.1 | 146.4 |  | 165.5 | 0.0 |  | 33.7 | 1347.9 |  | 184.8 | 0.4 |  | 0.0 | 3.0 |
| Czech Republic |  | 290.4 | 0.2 |  | 85.1 | 2053.5 |  | 1.3 | 0.0 |  | 0.0 | 0.0 |  | 0.0 | 0.0 |  | 705.1 | 24.5 |
| Peru |  | 24.6 | 0.3 |  | 188.6 | 3.4 |  | 0.0 | 0.0 |  | 506.3 | 1587.5 |  | 0.0 | 152.9 |  | 35.1 | 1.2 |
| Zimbabwe |  | 14.6 | 689.2 |  | 229.7 | 0.2 |  | 9.2 | 0.0 |  | 0.2 | 0.9 |  | 13.5 | 0.0 |  | 48.4 | 545.9 |
| Hungary |  | 202.6 | 0.0 |  | 1661.2 | 3610.4 |  | 3.2 | 0.0 |  | 4.0 | 3.4 |  | 3.7 | 0.0 |  | 871.0 | 30.1 |
| Afghanistan |  | 22.4 | 20.1 |  | 37.0 | 59.8 |  | 9.6 | 0.5 |  | 74.5 | 1253.9 |  | 0.1 | 66.8 |  | 399.6 | 21.9 |
| Azerbaijan |  | 38.9 | 0.2 |  | 24.0 | 53.2 |  | 0.1 | 0.1 |  | 17.1 | 1137.3 |  | 0.0 | 0.0 |  | 240.9 | 3.0 |
| Austria |  | 231.5 | 0.0 |  | 613.1 | 1973.5 |  | 1.4 | 0.0 |  | 0.0 | 0.0 |  | 0.3 | 2.1 |  | 322.2 | 5.4 |
| United Kingdom |  | 1361.3 | 6.6 |  | 0.0 | 0.0 |  | 0.0 | 0.0 |  | 0.0 | 0.0 |  | 0.0 | 0.3 |  | 2938.1 | 5082.5 |
| Nepal |  | 4.8 | 23.8 |  | 309.3 | 17.6 |  | 53.3 | 0.0 |  | 1256.3 | 2531.7 |  | 0.0 | 0.0 |  | 246.8 | 60.3 |
| Romania |  | 202.2 | 183.2 |  | 2341.4 | 3844.1 |  | 1.1 | 3.1 |  | 2.8 | 127.0 |  | 0.5 | 11.2 |  | 1093.0 | 190.3 |
| Cameroon |  | 0.0 | 0.0 |  | 180.6 | 37.5 |  | 16.8 | 1.4 |  | 54.3 | 1005.9 |  | 100.5 | 4.1 |  | 0.1 | 7.3 |
| Uruguay |  | 41.2 | 0.0 |  | 50.0 | 8.6 |  | 0.0 | 0.0 |  | 318.8 | 1168.4 |  | 19.8 | 18.4 |  | 70.3 | 0.0 |
| Mali |  | 0.0 | 6.4 |  | 44.0 | 129.5 |  | 153.9 | 3.3 |  | 180.3 | 998.4 |  | 77.1 | 5.9 |  | 2.1 | 5.0 |
| Serbia |  | 65.6 | 0.0 |  | 1268.3 | 924.8 |  | 0.4 | 1435.1 |  | 0.4 | 33.5 |  | 0.3 | 27.4 |  | 449.8 | 17.0 |
| Venezuela |  | 0.0 | 0.0 |  | 263.6 | 19.2 |  | 0.0 | 0.0 |  | 242.8 | 1228.7 |  | 99.0 | 3.0 |  | 0.0 | 0.0 |
| Uzbekistan |  | 23.0 | 0.1 |  | 39.8 | 1883.0 |  | 0.1 | 0.9 |  | 98.5 | 31.5 |  | 6.7 | 19.6 |  | 1180.8 | 52.7 |
| Ethiopia |  | 136.5 | 33.2 |  | 580.3 | 1222.5 |  | 54.5 | 43.4 |  | 0.1 | 22.7 |  | 239.3 | 307.0 |  | 179.6 | 150.7 |
| El Salvador |  | 0.0 | 0.0 |  | 127.9 | 1.9 |  | 0.0 | 0.0 |  | 12.5 | 754.7 |  | 28.3 | 0.0 |  | 0.2 | 0.0 |
| Bangladesh |  | 1.7 | 0.0 |  | 8.8 | 348.1 |  | 10.0 | 0.0 |  | 12920.2 | 13451.5 |  | 0.1 | 0.0 |  | 388.2 | 100.2 |
| New Zealand |  | 199.7 | 5.7 |  | 129.0 | 1022.2 |  | 0.0 | 0.0 |  | 0.0 | 0.0 |  | 0.0 | 0.0 |  | 193.6 | 63.4 |
| Bulgaria |  | 157.1 | 281.1 |  | 367.6 | 803.1 |  | 1.9 | 5.7 |  | 21.5 | 569.9 |  | 3.3 | 0.7 |  | 689.3 | 135.7 |
| Denmark |  | 817.1 | 14.9 |  | 0.0 | 397.1 |  | 0.0 | 0.0 |  | 0.0 | 0.0 |  | 0.0 | 0.0 |  | 1054.6 | 2007.5 |
| Cote d'Ivoire |  | 0.0 | 0.0 |  | 165.8 | 2.6 |  | 13.3 | 0.0 |  | 399.5 | 1114.6 |  | 5.5 | 0.0 |  | 0.0 | 0.0 |
| Colombia |  | 3.0 | 2.9 |  | 230.0 | 69.9 |  | 0.0 | 0.0 |  | 730.5 | 1201.5 |  | 54.9 | 210.7 |  | 9.9 | 21.2 |
| Kyrgyzstan |  | 27.2 | 0.0 |  | 76.9 | 798.5 |  | 0.0 | 0.0 |  | 17.8 | 13.6 |  | 0.2 | 1.2 |  | 220.7 | 0.2 |
| Chad |  | 0.0 | 0.0 |  | 29.1 | 5.5 |  | 67.2 | 0.2 |  | 13.4 | 271.6 |  | 49.7 | 0.1 |  | 0.0 | 345.8 |
| Zambia |  | 0.0 | 4.3 |  | 162.4 | 15.2 |  | 15.5 | 0.1 |  | 9.7 | 3.2 |  | 7.6 | 0.8 |  | 25.3 | 658.5 |
| Portugal |  | 12.4 | 0.0 |  | 186.3 | 548.7 |  | 0.0 | 0.0 |  | 39.4 | 181.8 |  | 0.0 | 16.9 |  | 52.0 | 0.0 |
| Myanmar |  | 0.7 | 5.1 |  | 115.3 | 49.3 |  | 42.3 | 0.9 |  | 7037.7 | 7627.8 |  | 0.8 | 0.0 |  | 30.9 | 1.5 |
| Yemen |  | 24.8 | 17.2 |  | 27.1 | 428.9 |  | 29.1 | 1.4 |  | 0.0 | 0.0 |  | 136.2 | 4.2 |  | 96.9 | 317.1 |
| Somalia |  | 0.5 | 0.0 |  | 60.3 | 3.3 |  | 0.3 | 0.0 |  | 0.0 | 545.4 |  | 37.2 | 0.2 |  | 1.3 | 0.8 |
| Angola |  | 0.0 | 0.0 |  | 105.7 | 0.0 |  | 29.8 | 0.0 |  | 4.6 | 511.7 |  | 0.4 | 0.0 |  | 0.0 | 77.8 |
| Paraguay |  | 0.2 | 0.0 |  | 166.2 | 59.8 |  | 0.0 | 0.0 |  | 46.5 | 640.5 |  | 8.2 | 2.7 |  | 48.0 | 0.1 |
| North Korea |  | 14.6 | 6.0 |  | 327.6 | 54.5 |  | 9.3 | 0.0 |  | 660.3 | 1382.3 |  | 4.8 | 15.7 |  | 26.2 | 0.0 |
| Libya |  | 25.7 | 1.3 |  | 1.6 | 477.4 |  | 4.4 | 18.4 |  | 0.0 | 0.0 |  | 0.0 | 0.0 |  | 70.8 | 18.3 |
| Belarus |  | 285.8 | 0.0 |  | 15.0 | 702.7 |  | 0.7 | 0.0 |  | 0.0 | 58.5 |  | 0.0 | 0.0 |  | 175.8 | 108.6 |
| Japan |  | 41.7 | 6.2 |  | 0.0 | 0.7 |  | 0.0 | 0.0 |  | 3450.6 | 3983.6 |  | 0.0 | 0.0 |  | 122.9 | 0.5 |
| Guatemala |  | 0.3 | 0.0 |  | 337.0 | 22.5 |  | 0.0 | 0.0 |  | 16.9 | 665.4 |  | 10.6 | 14.1 |  | 4.0 | 34.7 |
| Uganda |  | 0.5 | 41.2 |  | 298.8 | 158.6 |  | 152.3 | 69.3 |  | 55.6 | 642.8 |  | 87.3 | 1.7 |  | 7.0 | 27.2 |
| Vietnam |  | 0.4 | 5.8 |  | 378.0 | 1187.9 |  | 0.0 | 0.0 |  | 9815.8 | 9336.4 |  | 0.0 | 0.0 |  | 1.2 | 0.0 |
| Ecuador |  | 5.6 | 0.0 |  | 122.3 | 21.9 |  | 0.0 | 0.0 |  | 314.3 | 735.7 |  | 2.0 | 19.7 |  | 4.1 | 2.6 |
| Togo |  | 0.0 | 0.0 |  | 92.3 | 1.7 |  | 6.9 | 0.0 |  | 23.1 | 478.7 |  | 31.8 | 0.0 |  | 0.0 | 0.0 |
| Mozambique |  | 0.1 | 64.7 |  | 214.1 | 119.1 |  | 15.7 | 7.3 |  | 45.9 | 321.2 |  | 43.5 | 30.7 |  | 1.2 | 97.6 |
| Ghana |  | 0.0 | 0.0 |  | 226.5 | 69.3 |  | 26.4 | 0.0 |  | 67.2 | 618.2 |  | 47.8 | 0.0 |  | 0.0 | 0.0 |
| Sweden |  | 479.8 | 25.6 |  | 0.0 | 0.0 |  | 0.0 | 0.0 |  | 0.0 | 0.0 |  | 0.0 | 0.0 |  | 618.6 | 1388.8 |
| Macedonia |  | 18.8 | 0.0 |  | 39.2 | 213.6 |  | 0.0 | 0.2 |  | 7.2 | 219.8 |  | 0.0 | 0.9 |  | 78.0 | 0.4 |
| Iraq |  | 23.1 | 0.8 |  | 45.3 | 335.1 |  | 0.1 | 0.0 |  | 35.8 | 89.5 |  | 0.0 | 0.3 |  | 56.9 | 14.8 |
| Chile |  | 13.2 | 0.9 |  | 181.6 | 677.1 |  | 0.0 | 0.0 |  | 50.5 | 1.9 |  | 0.0 | 0.1 |  | 216.2 | 55.6 |
| Moldova |  | 37.8 | 29.3 |  | 321.9 | 304.9 |  | 1.3 | 0.0 |  | 0.1 | 257.5 |  | 0.0 | 0.0 |  | 179.0 | 218.9 |
| Senegal |  | 0.0 | 0.0 |  | 18.6 | 86.4 |  | 87.0 | 3.5 |  | 77.7 | 373.3 |  | 19.2 | 3.1 |  | 0.0 | 0.0 |
| Slovakia |  | 105.4 | 4.4 |  | 201.9 | 806.8 |  | 0.6 | 0.0 |  | 0.0 | 0.0 |  | 0.3 | 0.0 |  | 323.7 | 73.4 |
| Jordan |  | 10.3 | 0.9 |  | 17.5 | 281.7 |  | 0.0 | 0.0 |  | 0.0 | 0.0 |  | 0.1 | 0.8 |  | 6.5 | 0.2 |
| Nicaragua |  | 0.0 | 0.0 |  | 91.3 | 2.8 |  | 0.0 | 0.0 |  | 97.5 | 413.4 |  | 21.0 | 42.1 |  | 0.0 | 0.0 |
| Thailand |  | 0.0 | 214.1 |  | 917.5 | 7628.0 |  | 0.7 | 0.0 |  | 8776.7 | 2132.2 |  | 32.3 | 0.0 |  | 0.6 | 0.0 |
| Honduras |  | 0.0 | 0.0 |  | 107.3 | 7.5 |  | 0.0 | 0.0 |  | 8.9 | 346.6 |  | 13.9 | 16.6 |  | 0.7 | 1.9 |
| Switzerland |  | 76.3 | 0.0 |  | 58.7 | 547.8 |  | 0.0 | 0.0 |  | 3.9 | 0.0 |  | 0.4 | 0.0 |  | 169.3 | 0.0 |
| Tanzania |  | 0.5 | 699.6 |  | 519.4 | 182.0 |  | 53.9 | 0.3 |  | 153.0 | 199.0 |  | 101.6 | 0.5 |  | 17.7 | 0.2 |
| Saudi Arabia |  | 152.8 | 1716.8 |  | 10.5 | 55.8 |  | 9.4 | 36.8 |  | 0.0 | 0.0 |  | 97.8 | 51.4 |  | 1787.1 | 431.4 |
| Haiti |  | 0.0 | 0.0 |  | 53.6 | 0.7 |  | 0.0 | 0.0 |  | 47.8 | 333.7 |  | 16.8 | 0.1 |  | 0.0 | 0.0 |
| Benin |  | 0.0 | 0.0 |  | 102.2 | 30.0 |  | 13.2 | 0.0 |  | 21.6 | 330.3 |  | 27.5 | 1.6 |  | 0.1 | 7.9 |
| Namibia |  | 0.0 | 0.0 |  | 15.0 | 0.3 |  | 8.1 | 0.1 |  | 0.0 | 1.7 |  | 0.9 | 0.3 |  | 0.3 | 224.2 |
| Tajikistan |  | 4.7 | 0.0 |  | 15.7 | 39.4 |  | 0.1 | 0.2 |  | 18.8 | 11.0 |  | 0.5 | 266.6 |  | 76.1 | 0.4 |
| Israel |  | 5.0 | 0.0 |  | 16.2 | 201.9 |  | 0.0 | 0.0 |  | 0.0 | 0.0 |  | 1.3 | 38.1 |  | 17.9 | 0.0 |
| Malawi |  | 0.0 | 5.0 |  | 262.9 | 99.7 |  | 2.8 | 0.3 |  | 18.3 | 330.1 |  | 3.3 | 0.8 |  | 1.3 | 51.0 |
| Bolivia |  | 16.2 | 3.2 |  | 145.0 | 372.2 |  | 0.0 | 0.0 |  | 121.3 | 97.7 |  | 31.2 | 50.8 |  | 30.2 | 0.1 |
| Finland |  | 587.8 | 174.1 |  | 0.3 | 6.0 |  | 0.0 | 0.0 |  | 0.0 | 0.0 |  | 0.0 | 0.0 |  | 177.6 | 759.2 |
| Croatia |  | 26.4 | 0.0 |  | 582.1 | 967.8 |  | 0.9 | 0.6 |  | 0.0 | 0.0 |  | 0.3 | 6.7 |  | 196.1 | 3.4 |
| Congo, DRC |  | 0.0 | 1.3 |  | 318.2 | 17.6 |  | 14.8 | 22.9 |  | 158.7 | 39.5 |  | 21.5 | 9.0 |  | 0.6 | 586.7 |
| Rwanda |  | 0.0 | 5.1 |  | 14.1 | 1.4 |  | 0.7 | 0.8 |  | 5.4 | 193.1 |  | 22.7 | 1.6 |  | 1.6 | 3.5 |
| South Korea |  | 47.3 | 0.0 |  | 21.9 | 0.5 |  | 0.9 | 0.0 |  | 2167.7 | 2396.1 |  | 0.8 | 0.0 |  | 3.4 | 0.0 |
| Mauritania |  | 0.0 | 26.7 |  | 3.4 | 0.1 |  | 1.0 | 0.0 |  | 23.6 | 151.6 |  | 12.6 | 0.0 |  | 0.0 | 0.2 |
| Armenia |  | 12.8 | 0.0 |  | 3.4 | 109.8 |  | 0.0 | 0.0 |  | 0.6 | 78.3 |  | 0.0 | 0.0 |  | 42.0 | 0.3 |
| Tunisia |  | 55.5 | 12.0 |  | 0.0 | 0.0 |  | 0.0 | 0.0 |  | 0.0 | 69.4 |  | 0.3 | 38.6 |  | 332.1 | 388.1 |
| Lithuania |  | 164.5 | 8.3 |  | 1.0 | 110.1 |  | 0.0 | 0.0 |  | 0.0 | 0.0 |  | 0.0 | 0.0 |  | 222.1 | 389.1 |
| Ireland |  | 204.3 | 0.0 |  | 0.0 | 0.0 |  | 0.0 | 0.0 |  | 0.0 | 0.0 |  | 0.0 | 0.0 |  | 103.5 | 419.5 |
| Montenegro |  | 3.6 | 0.0 |  | 94.5 | 0.1 |  | 0.0 | 248.5 |  | 0.0 | 0.0 |  | 0.0 | 0.0 |  | 42.1 | 0.0 |
| Burundi |  | 0.0 | 7.5 |  | 36.1 | 8.5 |  | 2.2 | 2.3 |  | 13.6 | 141.6 |  | 11.4 | 4.6 |  | 1.8 | 0.0 |
| Turkmenistan |  | 7.6 | 2.0 |  | 6.3 | 75.0 |  | 0.2 | 0.0 |  | 25.8 | 20.9 |  | 0.3 | 0.4 |  | 367.1 | 406.2 |
| Cuba |  | 0.0 | 0.0 |  | 79.0 | 1.1 |  | 0.0 | 0.0 |  | 238.1 | 403.0 |  | 0.0 | 0.0 |  | 0.0 | 0.0 |
| Norway |  | 164.9 | 2.1 |  | 0.0 | 0.0 |  | 0.0 | 0.0 |  | 0.0 | 0.0 |  | 0.0 | 0.0 |  | 103.0 | 341.8 |
| Lebanon |  | 10.2 | 0.0 |  | 1.9 | 94.8 |  | 0.0 | 0.0 |  | 0.0 | 0.0 |  | 0.4 | 1.0 |  | 47.2 | 38.3 |
| Swaziland |  | 0.0 | 0.0 |  | 26.3 | 3.9 |  | 0.0 | 0.0 |  | 0.0 | 95.9 |  | 0.2 | 0.0 |  | 0.3 | 0.9 |
| Cambodia |  | 0.0 | 52.0 |  | 36.8 | 421.2 |  | 0.0 | 0.0 |  | 1502.1 | 1132.1 |  | 0.5 | 7.4 |  | 0.0 | 0.0 |
| Central African Republic |  | 0.0 | 0.0 |  | 28.5 | 1.5 |  | 5.2 | 0.2 |  | 15.6 | 123.7 |  | 9.8 | 0.2 |  | 0.0 | 5.4 |
| Belgium |  | 22.5 | 9.5 |  | 35.0 | 167.5 |  | 0.0 | 0.0 |  | 0.0 | 0.0 |  | 1.0 | 2.8 |  | 96.2 | 39.1 |
| Bosnia & Herzegovina |  | 10.2 | 7.1 |  | 185.3 | 229.8 |  | 0.0 | 49.6 |  | 0.0 | 0.0 |  | 0.0 | 14.8 |  | 50.1 | 8.1 |
| Botswana |  | 0.4 | 14.0 |  | 5.6 | 3.2 |  | 0.6 | 0.0 |  | 0.0 | 16.2 |  | 3.6 | 1.3 |  | 1.3 | 37.3 |
| Kuwait |  | 5.0 | 0.5 |  | 0.8 | 65.2 |  | 0.0 | 0.0 |  | 0.0 | 0.0 |  | 0.0 | 0.0 |  | 0.2 | 0.8 |
| The Gambia |  | 0.0 | 0.0 |  | 3.5 | 11.6 |  | 27.4 | 0.0 |  | 14.1 | 89.7 |  | 2.4 | 0.0 |  | 0.0 | 0.0 |
| West Bank |  | 0.7 | 0.0 |  | 5.1 | 35.5 |  | 0.0 | 0.0 |  | 0.0 | 0.0 |  | 0.6 | 25.5 |  | 3.6 | 0.0 |
| Netherlands |  | 35.6 | 2.9 |  | 33.9 | 108.9 |  | 0.0 | 0.0 |  | 0.0 | 0.0 |  | 0.0 | 0.0 |  | 156.2 | 163.2 |
| Georgia |  | 7.3 | 0.1 |  | 80.7 | 117.6 |  | 0.0 | 0.0 |  | 0.6 | 42.3 |  | 0.0 | 0.0 |  | 30.3 | 7.1 |
| Albania |  | 1.2 | 0.3 |  | 38.7 | 89.2 |  | 0.0 | 24.4 |  | 0.0 | 2.8 |  | 0.0 | 0.9 |  | 56.9 | 24.5 |
| Eritrea |  | 4.3 | 1.0 |  | 3.2 | 13.9 |  | 3.3 | 0.1 |  | 0.0 | 31.1 |  | 21.5 | 18.4 |  | 2.8 | 9.2 |
| Latvia |  | 49.5 | 4.1 |  | 0.7 | 21.6 |  | 0.0 | 0.0 |  | 0.0 | 0.0 |  | 0.0 | 0.0 |  | 84.0 | 146.8 |
| Dominican Republic |  | 0.0 | 0.0 |  | 7.5 | 0.1 |  | 0.0 | 0.0 |  | 198.0 | 244.8 |  | 2.6 | 0.1 |  | 0.0 | 0.0 |
| Timor-Leste |  | 0.0 | 0.0 |  | 25.0 | 1.5 |  | 0.0 | 0.0 |  | 18.4 | 78.2 |  | 0.0 | 0.0 |  | 0.0 | 0.0 |
| Guinea |  | 0.0 | 0.0 |  | 18.6 | 1.1 |  | 4.7 | 0.1 |  | 210.3 | 271.4 |  | 3.6 | 0.0 |  | 0.0 | 0.0 |
| Slovenia |  | 9.7 | 0.0 |  | 129.3 | 185.2 |  | 0.3 | 0.0 |  | 0.0 | 0.0 |  | 0.1 | 0.0 |  | 13.0 | 1.0 |
| Mongolia |  | 2.0 | 21.5 |  | 4.3 | 32.7 |  | 0.1 | 0.2 |  | 0.3 | 9.7 |  | 0.0 | 2.0 |  | 36.4 | 6.1 |
| Lesotho |  | 0.3 | 0.3 |  | 30.8 | 4.7 |  | 0.0 | 0.0 |  | 0.0 | 17.4 |  | 4.2 | 21.6 |  | 3.9 | 23.4 |
| Sri Lanka |  | 0.0 | 0.0 |  | 7.8 | 6.5 |  | 1.5 | 0.0 |  | 839.9 | 869.4 |  | 0.0 | 0.0 |  | 0.0 | 0.0 |
| Panama |  | 0.0 | 0.0 |  | 16.6 | 10.4 |  | 0.0 | 0.0 |  | 60.8 | 55.4 |  | 2.1 | 34.0 |  | 0.0 | 0.0 |
| Costa Rica |  | 0.0 | 0.0 |  | 5.8 | 0.2 |  | 0.0 | 0.0 |  | 80.4 | 101.4 |  | 0.4 | 1.2 |  | 0.0 | 0.0 |
| Guinea-Bissau |  | 0.0 | 0.0 |  | 4.8 | 0.3 |  | 4.2 | 0.0 |  | 27.5 | 54.4 |  | 2.4 | 0.0 |  | 0.0 | 0.0 |
| Bhutan |  | 0.8 | 0.0 |  | 9.2 | 12.2 |  | 1.7 | 0.2 |  | 27.6 | 33.7 |  | 0.1 | 0.0 |  | 2.0 | 9.1 |
| Gabon |  | 0.0 | 0.0 |  | 10.3 | 3.8 |  | 0.0 | 0.0 |  | 0.4 | 19.3 |  | 0.1 | 0.0 |  | 0.0 | 0.0 |
| Guyana |  | 0.0 | 0.0 |  | 2.8 | 4.9 |  | 0.0 | 0.0 |  | 138.9 | 147.6 |  | 0.1 | 0.5 |  | 0.0 | 0.0 |
| Malaysia |  | 0.0 | 0.0 |  | 19.2 | 129.4 |  | 0.0 | 0.0 |  | 786.9 | 687.0 |  | 0.2 | 0.0 |  | 0.0 | 0.0 |
| Estonia |  | 37.7 | 27.8 |  | 0.1 | 6.8 |  | 0.0 | 0.0 |  | 0.0 | 0.0 |  | 0.0 | 0.0 |  | 23.9 | 36.6 |
| Laos |  | 0.1 | 16.5 |  | 28.5 | 76.5 |  | 0.8 | 0.0 |  | 637.0 | 583.0 |  | 0.7 | 0.0 |  | 0.2 | 0.0 |
| Equatorial Guinea |  | 0.0 | 0.0 |  | 0.3 | 0.0 |  | 0.0 | 0.1 |  | 0.3 | 7.6 |  | 0.4 | 0.0 |  | 0.0 | 0.0 |
| Liberia |  | 0.0 | 0.0 |  | 0.9 | 2.7 |  | 0.1 | 0.1 |  | 109.1 | 111.6 |  | 0.0 | 0.0 |  | 0.0 | 0.0 |
| Luxembourg |  | 3.0 | 0.8 |  | 0.6 | 6.0 |  | 0.0 | 0.0 |  | 0.0 | 0.0 |  | 0.0 | 0.0 |  | 3.9 | 4.8 |
| Sierra Leone |  | 0.0 | 0.0 |  | 2.9 | 10.0 |  | 2.7 | 7.0 |  | 95.1 | 89.9 |  | 2.9 | 0.2 |  | 0.0 | 0.0 |
| Gaza Strip |  | 0.0 | 0.0 |  | 0.3 | 2.7 |  | 0.0 | 0.0 |  | 0.0 | 0.0 |  | 0.0 | 0.0 |  | 0.1 | 0.0 |
| Congo |  | 0.0 | 0.1 |  | 5.8 | 5.5 |  | 0.1 | 0.2 |  | 1.8 | 2.1 |  | 0.1 | 0.2 |  | 0.0 | 1.9 |
| Belize |  | 0.0 | 0.0 |  | 5.0 | 1.5 |  | 0.0 | 0.0 |  | 3.7 | 3.2 |  | 2.0 | 7.2 |  | 0.1 | 0.4 |
| Oman |  | 0.0 | 0.0 |  | 0.0 | 0.0 |  | 0.0 | 0.0 |  | 0.0 | 0.0 |  | 4.6 | 1.8 |  | 3.0 | 7.3 |
| United Arab Emirates |  | 0.0 | 0.0 |  | 0.0 | 0.0 |  | 0.0 | 0.0 |  | 0.0 | 0.0 |  | 1.3 | 0.5 |  | 0.4 | 2.1 |
| Suriname |  | 0.0 | 0.0 |  | 0.2 | 0.9 |  | 0.0 | 0.0 |  | 127.2 | 127.4 |  | 0.0 | 0.0 |  | 0.0 | 0.0 |
| Liechtenstein |  | 0.1 | 0.0 |  | 1.2 | 2.5 |  | 0.0 | 0.0 |  | 0.0 | 0.0 |  | 0.0 | 0.0 |  | 0.5 | 0.0 |
| San Marino |  | 0.0 | 0.0 |  | 0.1 | 0.9 |  | 0.0 | 0.0 |  | 0.0 | 0.0 |  | 0.0 | 0.0 |  | 0.2 | 0.0 |
| Andorra |  | 0.0 | 0.0 |  | 0.1 | 0.7 |  | 0.0 | 0.0 |  | 0.1 | 0.0 |  | 0.0 | 0.0 |  | 0.2 | 0.0 |
| Trinidad & Tobago |  | 0.0 | 0.0 |  | 0.6 | 0.0 |  | 0.0 | 0.0 |  | 2.6 | 3.4 |  | 0.0 | 0.0 |  | 0.0 | 0.0 |
| Jamaica |  | 0.0 | 0.0 |  | 0.4 | 0.0 |  | 0.0 | 0.0 |  | 0.0 | 0.7 |  | 0.0 | 0.0 |  | 0.0 | 0.0 |
| Brunei |  | 0.0 | 0.0 |  | 0.0 | 0.2 |  | 0.0 | 0.0 |  | 0.4 | 0.3 |  | 0.0 | 0.0 |  | 0.0 | 0.0 |
| Bahrain |  | 0.0 | 0.0 |  | 0.0 | 0.0 |  | 0.0 | 0.0 |  | 0.0 | 0.0 |  | 0.0 | 0.0 |  | 0.2 | 0.2 |
| Singapore |  | 0.0 | 0.0 |  | 0.0 | 0.0 |  | 0.0 | 0.0 |  | 0.0 | 0.1 |  | 0.0 | 0.0 |  | 0.0 | 0.0 |
| Isle of Man |  | 0.0 | 0.0 |  | 0.0 | 0.0 |  | 0.0 | 0.0 |  | 0.0 | 0.0 |  | 0.0 | 0.0 |  | 0.0 | 0.0 |
| French Guiana |  | 0.0 | 0.0 |  | 0.0 | 0.0 |  | 0.0 | 0.0 |  | 12.0 | 12.0 |  | 0.0 | 0.0 |  | 0.0 | 0.0 |
| Vatican City |  | 0.0 | 0.0 |  | 0.0 | 0.0 |  | 0.0 | 0.0 |  | 0.0 | 0.0 |  | 0.0 | 0.0 |  | 0.0 | 0.0 |
| Jersey |  | 0.0 | 0.0 |  | 0.0 | 0.0 |  | 0.0 | 0.0 |  | 0.0 | 0.0 |  | 0.0 | 0.0 |  | 0.0 | 0.0 |
| Gibraltar |  | 0.0 | 0.0 |  | 0.0 | 0.0 |  | 0.0 | 0.0 |  | 0.0 | 0.0 |  | 0.0 | 0.0 |  | 0.0 | 0.0 |
| Monaco |  | 0.0 | 0.0 |  | 0.0 | 0.0 |  | 0.0 | 0.0 |  | 0.0 | 0.0 |  | 0.0 | 0.0 |  | 0.0 | 0.0 |
| Puerto Rico |  | 0.0 | 0.0 |  | 0.4 | 0.4 |  | 0.0 | 0.0 |  | 0.0 | 0.0 |  | 0.0 | 0.0 |  | 0.0 | 0.0 |
| Papua New Guinea |  | 0.0 | 0.0 |  | 0.1 | 0.0 |  | 0.0 | 0.0 |  | 0.9 | 0.0 |  | 0.0 | 0.8 |  | 0.0 | 0.0 |
| Madagascar |  | 0.0 | 0.0 |  | 27.3 | 0.0 |  | 0.0 | 0.0 |  | 619.7 | 308.3 |  | 0.0 | 0.0 |  | 2.1 | 332.5 |
