# Supplementary material for: Transformative optimisation of agricultural land use to meet future food demands
Source: PeerJ. 2013 Oct 24;1:e188. doi: 10.7717/peerj.188 (PMC3817586; doi:10.7717/peerj.188)
Supplement: Table S6 [file peerj-01-188-s008.docx]

**Table S6. Potential annual revenue generated from oilseed crops in 167 countries under current and optimal land-use allocation options (all values in 10^6^ US$/y). Calculations were based on producer prices of each crop averaged among all producer countries over the period 2000-2007 (Source for price data: FAOSTAT. Statistical Databases and Data-Sets, Rome, Italy, Food and Agriculture Organization of the United Nations, 2010).**

|  |  | Soy | |  | Cottonseed | |  | Rapeseed | |  | Sunflower seed | |  | Groundnut | |  | Oil palm | |
| --- | --- | --- | --- | --- | --- | --- | --- | --- | --- | --- | --- | --- | --- | --- | --- | --- | --- | --- |
| Country |  | Current | Optimal |  | Current | Optimal |  | Current | Optimal |  | Current | Optimal |  | Current | Optimal |  | Current | Optimal |
| United States |  | 28084.3 | 31920.1 |  | 1898.1 | 406.4 |  | 221.9 | 214.6 |  | 551.3 | 62.7 |  | 1180.3 | 1654.8 |  | 0.0 | 0.0 |
| Canada |  | 1167.8 | 4297.4 |  | 0.0 | 0.0 |  | 2137.6 | 600.7 |  | 46.3 | 29.4 |  | 0.0 | 0.0 |  | 0.0 | 0.0 |
| India |  | 2190.2 | 6509.7 |  | 927.3 | 57.4 |  | 1499.2 | 2796.8 |  | 284.1 | 857.9 |  | 4536.5 | 376.8 |  | 0.0 | 0.0 |
| Argentina |  | 8071.3 | 11217.7 |  | 98.7 | 2.1 |  | 1.7 | 0.1 |  | 1624.7 | 1.0 |  | 411.5 | 0.2 |  | 0.1 | 0.0 |
| Australia |  | 77.8 | 694.4 |  | 566.9 | 1120.7 |  | 658.5 | 203.2 |  | 58.0 | 35.7 |  | 54.5 | 24.8 |  | 0.0 | 0.0 |
| Pakistan |  | 0.0 | 1764.6 |  | 1023.3 | 1.3 |  | 104.2 | 21.7 |  | 57.1 | 12.0 |  | 84.5 | 0.6 |  | 0.0 | 0.0 |
| Russia |  | 205.5 | 1572.4 |  | 4.6 | 32.8 |  | 89.0 | 10.0 |  | 1354.5 | 420.5 |  | 0.0 | 3.5 |  | 0.0 | 0.0 |
| Spain |  | 4.2 | 659.0 |  | 67.2 | 4.1 |  | 20.6 | 63.7 |  | 313.9 | 1.8 |  | 0.2 | 3.9 |  | 0.0 | 0.0 |
| Brazil |  | 12247.3 | 12900.0 |  | 297.4 | 9.5 |  | 0.0 | 127.8 |  | 52.5 | 0.4 |  | 143.6 | 0.0 |  | 28.3 | 20.2 |
| Thailand |  | 104.6 | 35.9 |  | 10.3 | 2.1 |  | 0.0 | 0.0 |  | 7.3 | 0.1 |  | 96.4 | 2.0 |  | 321.5 | 767.6 |
| Cote d'Ivoire |  | 0.2 | 55.7 |  | 66.0 | 71.0 |  | 0.0 | 0.0 |  | 0.0 | 0.0 |  | 109.1 | 0.0 |  | 121.5 | 404.8 |
| Egypt |  | 15.6 | 536.3 |  | 171.5 | 1.8 |  | 0.0 | 0.0 |  | 24.0 | 17.8 |  | 160.2 | 4.6 |  | 0.0 | 0.0 |
| Turkey |  | 25.2 | 396.6 |  | 449.2 | 14.7 |  | 0.0 | 558.5 |  | 258.7 | 8.5 |  | 65.1 | 0.2 |  | 0.0 | 0.0 |
| Ukraine |  | 36.7 | 1372.3 |  | 0.0 | 0.0 |  | 32.5 | 7.6 |  | 1131.7 | 1.0 |  | 0.0 | 0.0 |  | 0.0 | 0.0 |
| Uzbekistan |  | 0.0 | 15.6 |  | 656.5 | 261.5 |  | 0.0 | 6.6 |  | 7.7 | 549.4 |  | 13.7 | 1.6 |  | 0.0 | 0.0 |
| Romania |  | 51.8 | 557.7 |  | 0.0 | 0.0 |  | 15.4 | 24.1 |  | 372.9 | 12.3 |  | 0.6 | 0.0 |  | 0.0 | 0.0 |
| Iran |  | 90.1 | 375.7 |  | 131.0 | 7.4 |  | 0.2 | 1.5 |  | 34.0 | 2.8 |  | 0.0 | 18.1 |  | 0.0 | 0.0 |
| France |  | 118.4 | 679.6 |  | 0.0 | 0.0 |  | 1017.9 | 1218.7 |  | 615.1 | 2.6 |  | 0.0 | 0.0 |  | 0.0 | 0.0 |
| Paraguay |  | 946.2 | 1161.9 |  | 31.0 | 0.0 |  | 0.0 | 0.0 |  | 19.5 | 1.3 |  | 23.5 | 0.0 |  | 4.3 | 0.0 |
| Ethiopia |  | 1.0 | 180.0 |  | 14.1 | 0.1 |  | 8.1 | 0.0 |  | 0.0 | 0.0 |  | 19.2 | 0.0 |  | 0.0 | 0.0 |
| Others (below) |  | 7561.1 | 20881.8 |  | 4368.3 | 4745.7 |  | 5663.9 | 4394.7 |  | 2102.2 | 1452.8 |  | 16062.8 | 1253.6 |  | 10215.4 | 11646.3 |
| Benin |  | 2.7 | 93.2 |  | 57.9 | 1.7 |  | 0.0 | 0.0 |  | 0.0 | 0.0 |  | 46.2 | 0.0 |  | 12.6 | 151.4 |
| Kazakhstan |  | 7.4 | 245.1 |  | 75.0 | 13.8 |  | 5.1 | 0.8 |  | 50.4 | 0.6 |  | 0.3 | 0.2 |  | 0.0 | 0.0 |
| Indonesia |  | 316.2 | 325.0 |  | 0.0 | 1.0 |  | 0.0 | 0.0 |  | 0.0 | 0.0 |  | 877.3 | 1.2 |  | 3544.3 | 4532.8 |
| Colombia |  | 34.5 | 72.9 |  | 26.4 | 8.8 |  | 0.0 | 0.0 |  | 0.0 | 0.0 |  | 0.2 | 0.2 |  | 181.0 | 268.2 |
| Zimbabwe |  | 30.0 | 226.9 |  | 37.9 | 0.6 |  | 0.0 | 0.0 |  | 7.5 | 15.7 |  | 64.9 | 0.0 |  | 0.0 | 0.0 |
| Greece |  | 2.4 | 229.8 |  | 189.9 | 67.0 |  | 0.2 | 0.0 |  | 13.4 | 5.3 |  | 0.8 | 0.1 |  | 0.0 | 0.0 |
| Bolivia |  | 415.1 | 575.3 |  | 14.3 | 0.1 |  | 0.0 | 0.3 |  | 43.3 | 0.5 |  | 18.5 | 3.0 |  | 0.0 | 0.0 |
| Cameroon |  | 5.1 | 75.7 |  | 39.9 | 28.0 |  | 0.0 | 0.0 |  | 0.0 | 0.0 |  | 153.9 | 0.5 |  | 64.8 | 234.1 |
| Uganda |  | 73.1 | 242.2 |  | 12.7 | 0.1 |  | 0.0 | 0.0 |  | 3.1 | 16.1 |  | 97.7 | 0.0 |  | 1.1 | 1.8 |
| Italy |  | 367.2 | 502.5 |  | 0.0 | 0.0 |  | 17.9 | 58.6 |  | 110.6 | 5.0 |  | 0.0 | 0.0 |  | 0.0 | 0.0 |
| Portugal |  | 0.3 | 7.6 |  | 0.1 | 0.2 |  | 0.3 | 2.3 |  | 9.1 | 0.0 |  | 0.0 | 62.4 |  | 0.0 | 0.0 |
| Hungary |  | 16.7 | 296.9 |  | 0.0 | 0.0 |  | 55.3 | 54.8 |  | 243.3 | 17.4 |  | 0.0 | 0.0 |  | 0.0 | 0.0 |
| Uruguay |  | 17.2 | 96.7 |  | 0.0 | 0.0 |  | 0.0 | 0.5 |  | 31.1 | 5.4 |  | 0.9 | 0.0 |  | 0.0 | 0.0 |
| Togo |  | 0.1 | 13.2 |  | 29.6 | 16.0 |  | 0.0 | 0.0 |  | 0.0 | 0.0 |  | 21.5 | 2.8 |  | 7.6 | 80.0 |
| Venezuela |  | 0.3 | 66.2 |  | 6.8 | 0.0 |  | 0.0 | 0.0 |  | 1.5 | 0.0 |  | 0.0 | 0.0 |  | 11.1 | 0.9 |
| Ecuador |  | 29.0 | 3.2 |  | 2.5 | 0.7 |  | 0.0 | 0.0 |  | 0.0 | 0.0 |  | 9.9 | 2.5 |  | 123.6 | 203.8 |
| Serbia |  | 72.1 | 158.2 |  | 0.0 | 0.0 |  | 3.8 | 19.8 |  | 101.9 | 42.7 |  | 0.1 | 0.0 |  | 0.0 | 0.0 |
| Zambia |  | 7.5 | 99.7 |  | 14.1 | 0.3 |  | 0.0 | 0.0 |  | 4.5 | 0.1 |  | 34.0 | 0.0 |  | 0.9 | 0.7 |
| Bangladesh |  | 0.4 | 5.0 |  | 7.1 | 115.8 |  | 79.2 | 30.1 |  | 0.3 | 2.3 |  | 28.7 | 1.8 |  | 0.0 | 0.0 |
| Bulgaria |  | 3.7 | 132.2 |  | 3.2 | 1.1 |  | 4.8 | 115.8 |  | 194.8 | 2.4 |  | 7.8 | 0.0 |  | 0.0 | 0.0 |
| Azerbaijan |  | 1.8 | 31.4 |  | 19.5 | 1.5 |  | 0.0 | 0.0 |  | 3.4 | 0.2 |  | 0.0 | 27.6 |  | 0.0 | 0.0 |
| Syria |  | 6.7 | 79.8 |  | 376.2 | 283.9 |  | 0.0 | 9.0 |  | 7.9 | 89.3 |  | 40.5 | 4.0 |  | 0.0 | 0.0 |
| South Africa |  | 76.5 | 512.5 |  | 21.7 | 0.0 |  | 0.0 | 0.0 |  | 245.3 | 0.2 |  | 136.1 | 0.3 |  | 0.0 | 0.0 |
| Turkmenistan |  | 0.4 | 15.6 |  | 213.7 | 185.1 |  | 0.0 | 9.0 |  | 0.4 | 17.6 |  | 0.3 | 20.4 |  | 0.0 | 0.0 |
| Peru |  | 0.0 | 61.1 |  | 31.9 | 0.3 |  | 0.0 | 0.3 |  | 0.0 | 0.0 |  | 2.2 | 0.1 |  | 0.4 | 0.7 |
| Belarus |  | 0.9 | 3.1 |  | 0.0 | 0.0 |  | 20.6 | 4.4 |  | 10.7 | 51.2 |  | 0.0 | 0.0 |  | 0.0 | 0.0 |
| Papua New Guinea |  | 0.0 | 1.0 |  | 0.0 | 0.0 |  | 0.0 | 0.0 |  | 0.0 | 0.0 |  | 1.2 | 22.3 |  | 60.6 | 62.0 |
| The Gambia |  | 0.0 | 0.0 |  | 0.2 | 0.5 |  | 0.0 | 0.0 |  | 0.0 | 0.0 |  | 73.2 | 3.9 |  | 3.2 | 94.3 |
| Guatemala |  | 16.1 | 12.8 |  | 0.7 | 0.1 |  | 0.0 | 0.1 |  | 0.0 | 0.0 |  | 1.5 | 0.0 |  | 32.5 | 56.9 |
| Afghanistan |  | 0.4 | 3.9 |  | 31.0 | 29.5 |  | 0.9 | 0.3 |  | 0.7 | 12.2 |  | 0.2 | 1.4 |  | 0.0 | 0.0 |
| Georgia |  | 2.6 | 20.8 |  | 1.0 | 0.0 |  | 0.0 | 2.7 |  | 7.3 | 0.1 |  | 0.0 | 0.0 |  | 0.0 | 0.0 |
| Liberia |  | 1.8 | 1.5 |  | 0.9 | 1.5 |  | 0.0 | 0.0 |  | 0.0 | 0.0 |  | 6.4 | 0.6 |  | 21.4 | 39.8 |
| Kyrgyzstan |  | 0.1 | 2.3 |  | 21.3 | 29.0 |  | 0.1 | 0.0 |  | 14.1 | 17.2 |  | 0.8 | 0.0 |  | 0.0 | 0.0 |
| Germany |  | 1.2 | 38.9 |  | 0.0 | 0.0 |  | 956.5 | 955.7 |  | 28.0 | 2.4 |  | 0.0 | 0.0 |  | 0.0 | 0.0 |
| Swaziland |  | 0.3 | 5.9 |  | 1.8 | 0.3 |  | 0.0 | 0.0 |  | 0.3 | 1.2 |  | 4.5 | 10.2 |  | 0.0 | 0.0 |
| Croatia |  | 31.3 | 57.9 |  | 0.0 | 0.0 |  | 10.4 | 11.9 |  | 20.7 | 1.5 |  | 0.0 | 0.0 |  | 0.0 | 0.0 |
| Honduras |  | 2.2 | 4.5 |  | 0.1 | 0.0 |  | 0.0 | 0.0 |  | 0.0 | 0.0 |  | 1.0 | 0.0 |  | 56.4 | 64.1 |
| Iraq |  | 1.1 | 0.8 |  | 3.9 | 1.8 |  | 0.0 | 0.3 |  | 2.5 | 12.7 |  | 0.6 | 1.2 |  | 0.0 | 0.0 |
| Mexico |  | 43.6 | 55.7 |  | 63.8 | 59.2 |  | 0.0 | 43.9 |  | 0.1 | 11.0 |  | 96.0 | 0.0 |  | 11.8 | 53.2 |
| Tajikistan |  | 0.0 | 0.1 |  | 71.6 | 62.4 |  | 0.0 | 0.9 |  | 1.3 | 17.0 |  | 0.5 | 0.5 |  | 0.0 | 0.0 |
| Nepal |  | 7.8 | 15.9 |  | 0.1 | 0.7 |  | 4.1 | 1.9 |  | 0.0 | 0.1 |  | 0.8 | 1.1 |  | 0.0 | 0.0 |
| Bosnia & Herzegovina |  | 4.1 | 3.9 |  | 0.0 | 0.0 |  | 1.3 | 11.8 |  | 4.0 | 0.3 |  | 0.0 | 0.0 |  | 0.0 | 0.0 |
| Philippines |  | 0.5 | 14.2 |  | 0.7 | 1.4 |  | 0.0 | 0.0 |  | 0.0 | 0.0 |  | 21.3 | 12.7 |  | 0.0 | 0.0 |
| North Korea |  | 126.7 | 135.2 |  | 6.4 | 0.6 |  | 0.0 | 1.9 |  | 0.4 | 2.0 |  | 0.7 | 0.0 |  | 0.0 | 0.0 |
| Slovakia |  | 5.4 | 28.5 |  | 0.0 | 0.0 |  | 45.9 | 61.3 |  | 42.8 | 9.5 |  | 0.0 | 0.0 |  | 0.0 | 0.0 |
| Macedonia |  | 0.7 | 6.0 |  | 1.7 | 0.4 |  | 1.0 | 4.7 |  | 4.2 | 1.7 |  | 0.1 | 0.0 |  | 0.0 | 0.0 |
| Botswana |  | 1.4 | 7.1 |  | 0.6 | 5.2 |  | 0.0 | 0.0 |  | 3.3 | 0.2 |  | 2.3 | 0.0 |  | 0.0 | 0.0 |
| Haiti |  | 0.0 | 0.0 |  | 0.3 | 0.0 |  | 0.0 | 0.0 |  | 0.0 | 0.0 |  | 15.0 | 15.8 |  | 0.7 | 4.4 |
| Dominican Republic |  | 0.0 | 0.0 |  | 0.0 | 0.0 |  | 0.0 | 0.0 |  | 0.0 | 0.0 |  | 3.8 | 3.6 |  | 10.2 | 14.5 |
| Laos |  | 3.7 | 1.1 |  | 4.3 | 18.3 |  | 0.1 | 0.0 |  | 1.0 | 0.1 |  | 16.1 | 0.0 |  | 3.3 | 12.9 |
| Bhutan |  | 1.0 | 5.5 |  | 0.0 | 0.0 |  | 3.8 | 2.6 |  | 0.0 | 0.0 |  | 0.0 | 0.6 |  | 0.0 | 0.0 |
| Cambodia |  | 19.7 | 22.9 |  | 0.5 | 7.2 |  | 0.0 | 0.0 |  | 0.1 | 0.0 |  | 12.6 | 3.5 |  | 1.6 | 4.5 |
| Congo |  | 0.1 | 3.7 |  | 0.1 | 0.5 |  | 0.0 | 0.0 |  | 0.0 | 0.0 |  | 36.3 | 32.6 |  | 1.6 | 4.8 |
| Equatorial Guinea |  | 0.0 | 0.2 |  | 0.0 | 0.0 |  | 0.0 | 0.0 |  | 0.0 | 0.0 |  | 2.3 | 0.0 |  | 9.0 | 14.4 |
| Switzerland |  | 6.2 | 19.2 |  | 0.0 | 0.0 |  | 11.7 | 6.5 |  | 4.5 | 0.0 |  | 0.0 | 0.0 |  | 0.0 | 0.0 |
| Montenegro |  | 5.7 | 17.1 |  | 0.0 | 0.0 |  | 0.4 | 0.2 |  | 8.7 | 0.7 |  | 0.0 | 0.0 |  | 0.0 | 0.0 |
| Czech Republic |  | 3.7 | 0.5 |  | 0.0 | 0.0 |  | 181.4 | 200.4 |  | 16.7 | 4.1 |  | 0.0 | 0.0 |  | 0.0 | 0.0 |
| Armenia |  | 0.2 | 4.8 |  | 2.1 | 0.2 |  | 0.0 | 0.5 |  | 0.8 | 0.0 |  | 0.0 | 0.1 |  | 0.0 | 0.0 |
| Sierra Leone |  | 0.0 | 0.0 |  | 0.5 | 1.7 |  | 0.0 | 0.0 |  | 0.0 | 0.0 |  | 19.1 | 3.1 |  | 16.0 | 33.3 |
| El Salvador |  | 1.5 | 3.2 |  | 0.6 | 0.0 |  | 0.0 | 0.0 |  | 0.0 | 0.0 |  | 0.1 | 0.0 |  | 2.1 | 3.4 |
| Mozambique |  | 1.3 | 17.2 |  | 11.4 | 3.6 |  | 0.0 | 0.0 |  | 9.2 | 70.5 |  | 69.2 | 2.1 |  | 0.0 | 0.0 |
| Chile |  | 0.0 | 0.0 |  | 0.1 | 0.0 |  | 17.2 | 21.6 |  | 2.5 | 0.0 |  | 0.0 | 0.0 |  | 0.0 | 0.0 |
| Albania |  | 1.5 | 3.7 |  | 0.2 | 0.2 |  | 0.1 | 0.6 |  | 2.5 | 0.9 |  | 0.0 | 0.8 |  | 0.0 | 0.0 |
| Poland |  | 0.4 | 0.3 |  | 0.0 | 0.0 |  | 294.8 | 298.7 |  | 2.8 | 0.5 |  | 0.0 | 0.0 |  | 0.0 | 0.0 |
| Costa Rica |  | 0.1 | 0.5 |  | 0.0 | 0.7 |  | 0.0 | 0.0 |  | 0.0 | 0.0 |  | 0.7 | 0.0 |  | 51.1 | 52.1 |
| Slovenia |  | 3.2 | 5.9 |  | 0.0 | 0.0 |  | 0.6 | 0.0 |  | 1.6 | 0.8 |  | 0.0 | 0.0 |  | 0.0 | 0.0 |
| Belgium |  | 0.0 | 2.9 |  | 0.0 | 0.0 |  | 4.5 | 2.6 |  | 0.5 | 0.8 |  | 0.0 | 0.0 |  | 0.0 | 0.0 |
| Panama |  | 0.0 | 1.2 |  | 0.0 | 0.0 |  | 0.0 | 0.0 |  | 0.0 | 0.0 |  | 0.0 | 0.0 |  | 2.6 | 2.6 |
| Lithuania |  | 0.1 | 0.0 |  | 0.0 | 0.0 |  | 18.5 | 17.6 |  | 0.5 | 2.8 |  | 0.0 | 0.0 |  | 0.0 | 0.0 |
| Somalia |  | 0.0 | 0.5 |  | 0.4 | 0.4 |  | 0.0 | 0.0 |  | 0.0 | 0.7 |  | 0.2 | 0.0 |  | 0.0 | 0.0 |
| Austria |  | 20.0 | 40.9 |  | 0.0 | 0.0 |  | 56.3 | 64.0 |  | 27.8 | 0.0 |  | 0.0 | 0.0 |  | 0.0 | 0.0 |
| Saudi Arabia |  | 0.0 | 0.0 |  | 0.0 | 0.0 |  | 0.0 | 0.0 |  | 0.0 | 0.0 |  | 0.0 | 0.7 |  | 0.0 | 0.0 |
| Lesotho |  | 0.6 | 4.0 |  | 0.0 | 0.1 |  | 0.0 | 0.0 |  | 2.7 | 0.6 |  | 1.1 | 0.3 |  | 0.0 | 0.0 |
| Mongolia |  | 0.8 | 1.3 |  | 0.0 | 0.2 |  | 0.1 | 0.1 |  | 0.6 | 0.5 |  | 0.0 | 0.0 |  | 0.0 | 0.0 |
| Eritrea |  | 0.0 | 1.5 |  | 0.1 | 0.2 |  | 0.0 | 0.0 |  | 0.0 | 0.2 |  | 2.9 | 1.6 |  | 0.0 | 0.0 |
| Latvia |  | 0.0 | 0.0 |  | 0.0 | 0.0 |  | 4.7 | 4.7 |  | 0.2 | 0.5 |  | 0.0 | 0.0 |  | 0.0 | 0.0 |
| Netherlands |  | 0.0 | 1.1 |  | 0.0 | 0.0 |  | 4.3 | 3.6 |  | 0.0 | 0.0 |  | 0.0 | 0.0 |  | 0.0 | 0.0 |
| Tunisia |  | 0.0 | 0.0 |  | 0.9 | 5.9 |  | 0.9 | 0.2 |  | 4.0 | 0.0 |  | 0.1 | 0.0 |  | 0.0 | 0.0 |
| Yemen |  | 0.0 | 0.0 |  | 7.4 | 7.3 |  | 0.0 | 0.0 |  | 0.0 | 0.0 |  | 0.0 | 0.2 |  | 0.0 | 0.0 |
| Suriname |  | 0.0 | 0.1 |  | 0.0 | 0.0 |  | 0.0 | 0.0 |  | 0.0 | 0.0 |  | 0.4 | 0.5 |  | 0.0 | 0.0 |
| Jordan |  | 0.1 | 0.0 |  | 7.4 | 9.0 |  | 0.0 | 0.0 |  | 0.7 | 0.1 |  | 1.0 | 0.1 |  | 0.0 | 0.0 |
| Finland |  | 0.0 | 0.0 |  | 0.0 | 0.0 |  | 54.9 | 55.0 |  | 0.0 | 0.0 |  | 0.0 | 0.0 |  | 0.0 | 0.0 |
| Belize |  | 1.2 | 1.2 |  | 0.0 | 0.0 |  | 0.0 | 0.0 |  | 0.0 | 0.0 |  | 0.0 | 0.0 |  | 0.0 | 0.0 |
| Luxembourg |  | 0.0 | 0.0 |  | 0.0 | 0.0 |  | 1.3 | 1.4 |  | 0.1 | 0.0 |  | 0.0 | 0.0 |  | 0.0 | 0.0 |
| Estonia |  | 0.1 | 0.1 |  | 0.0 | 0.0 |  | 8.4 | 8.4 |  | 0.0 | 0.0 |  | 0.0 | 0.0 |  | 0.0 | 0.0 |
| Liechtenstein |  | 0.0 | 0.2 |  | 0.0 | 0.0 |  | 0.2 | 0.1 |  | 0.0 | 0.0 |  | 0.0 | 0.0 |  | 0.0 | 0.0 |
| Namibia |  | 0.0 | 0.1 |  | 0.5 | 0.7 |  | 0.0 | 0.0 |  | 0.0 | 0.0 |  | 0.3 | 0.0 |  | 0.0 | 0.0 |
| Kuwait |  | 0.0 | 0.0 |  | 0.0 | 0.0 |  | 0.0 | 0.0 |  | 0.0 | 0.0 |  | 0.0 | 0.0 |  | 0.0 | 0.0 |
| San Marino |  | 0.1 | 0.1 |  | 0.0 | 0.0 |  | 0.0 | 0.0 |  | 0.0 | 0.0 |  | 0.0 | 0.0 |  | 0.0 | 0.0 |
| Andorra |  | 0.0 | 0.0 |  | 0.0 | 0.0 |  | 0.0 | 0.1 |  | 0.1 | 0.0 |  | 0.0 | 0.0 |  | 0.0 | 0.0 |
| Gibraltar |  | 0.0 | 0.0 |  | 0.0 | 0.0 |  | 0.0 | 0.0 |  | 0.0 | 0.0 |  | 0.0 | 0.0 |  | 0.0 | 0.0 |
| Vatican City |  | 0.0 | 0.0 |  | 0.0 | 0.0 |  | 0.0 | 0.0 |  | 0.0 | 0.0 |  | 0.0 | 0.0 |  | 0.0 | 0.0 |
| Monaco |  | 0.0 | 0.0 |  | 0.0 | 0.0 |  | 0.0 | 0.0 |  | 0.0 | 0.0 |  | 0.0 | 0.0 |  | 0.0 | 0.0 |
| United Kingdom |  | 0.0 | 0.0 |  | 0.0 | 0.0 |  | 463.8 | 463.8 |  | 0.0 | 0.0 |  | 0.0 | 0.0 |  | 0.0 | 0.0 |
| Denmark |  | 0.0 | 0.0 |  | 0.0 | 0.0 |  | 104.3 | 104.3 |  | 0.0 | 0.0 |  | 0.0 | 0.0 |  | 0.0 | 0.0 |
| Singapore |  | 0.0 | 0.0 |  | 0.0 | 0.0 |  | 0.0 | 0.0 |  | 0.0 | 0.0 |  | 0.0 | 0.0 |  | 20.6 | 20.6 |
| Sweden |  | 0.0 | 0.0 |  | 0.0 | 0.0 |  | 43.0 | 43.0 |  | 0.0 | 0.0 |  | 0.0 | 0.0 |  | 0.0 | 0.0 |
| Solomon Is. |  | 0.0 | 0.0 |  | 0.0 | 0.0 |  | 0.0 | 0.0 |  | 0.0 | 0.0 |  | 0.0 | 0.0 |  | 11.4 | 11.4 |
| Sao Tome & Principe |  | 0.0 | 0.0 |  | 0.0 | 0.0 |  | 0.0 | 0.0 |  | 0.0 | 0.0 |  | 0.0 | 0.0 |  | 3.6 | 3.6 |
| Libya |  | 0.0 | 0.0 |  | 0.0 | 0.0 |  | 0.0 | 0.0 |  | 0.0 | 0.0 |  | 27.6 | 27.6 |  | 0.0 | 0.0 |
| Brunei |  | 0.0 | 0.0 |  | 0.0 | 0.0 |  | 0.0 | 0.0 |  | 0.0 | 0.0 |  | 0.0 | 0.0 |  | 2.6 | 2.6 |
| Norway |  | 0.0 | 0.0 |  | 0.0 | 0.0 |  | 6.1 | 6.1 |  | 0.0 | 0.0 |  | 0.0 | 0.0 |  | 0.0 | 0.0 |
| New Zealand |  | 0.0 | 0.0 |  | 0.0 | 0.0 |  | 4.9 | 4.9 |  | 0.0 | 0.0 |  | 0.0 | 0.0 |  | 0.0 | 0.0 |
| Cuba |  | 0.0 | 0.0 |  | 0.0 | 0.0 |  | 0.0 | 0.0 |  | 0.0 | 0.0 |  | 10.0 | 10.0 |  | 0.0 | 0.0 |
| Ireland |  | 0.0 | 0.0 |  | 0.0 | 0.0 |  | 4.1 | 4.1 |  | 0.0 | 0.0 |  | 0.0 | 0.0 |  | 0.0 | 0.0 |
| Jamaica |  | 0.0 | 0.0 |  | 0.0 | 0.0 |  | 0.0 | 0.0 |  | 0.0 | 0.0 |  | 2.6 | 2.6 |  | 0.0 | 0.0 |
| Isle of Man |  | 0.0 | 0.0 |  | 0.0 | 0.0 |  | 0.0 | 0.0 |  | 0.0 | 0.0 |  | 0.0 | 0.0 |  | 0.0 | 0.0 |
| Djibouti |  | 0.0 | 0.0 |  | 0.0 | 0.0 |  | 0.0 | 0.0 |  | 0.0 | 0.0 |  | 0.0 | 0.0 |  | 0.0 | 0.0 |
| Gaza Strip |  | 0.0 | 0.0 |  | 0.2 | 0.2 |  | 0.0 | 0.0 |  | 0.0 | 0.0 |  | 0.1 | 0.0 |  | 0.0 | 0.0 |
| Guyana |  | 0.0 | 0.1 |  | 0.0 | 0.0 |  | 0.0 | 0.2 |  | 0.0 | 0.1 |  | 3.1 | 2.7 |  | 0.0 | 0.0 |
| Mauritania |  | 0.0 | 0.0 |  | 0.0 | 0.1 |  | 0.0 | 0.0 |  | 0.0 | 0.0 |  | 2.6 | 2.4 |  | 0.0 | 0.0 |
| Algeria |  | 0.0 | 0.0 |  | 0.0 | 0.0 |  | 17.8 | 25.1 |  | 0.2 | 0.3 |  | 9.2 | 1.4 |  | 0.0 | 0.0 |
| Burundi |  | 2.6 | 8.4 |  | 0.6 | 0.0 |  | 0.0 | 0.0 |  | 0.1 | 0.0 |  | 6.3 | 0.0 |  | 0.0 | 0.4 |
| Malaysia |  | 0.0 | 0.0 |  | 0.0 | 0.0 |  | 0.0 | 0.0 |  | 0.0 | 0.0 |  | 10.4 | 4.2 |  | 4997.6 | 5003.0 |
| Kenya |  | 2.2 | 7.9 |  | 6.4 | 0.2 |  | 0.0 | 0.1 |  | 5.0 | 30.4 |  | 26.4 | 0.5 |  | 0.0 | 0.0 |
| Moldova |  | 4.3 | 99.0 |  | 0.0 | 0.0 |  | 0.8 | 0.0 |  | 101.1 | 5.8 |  | 0.0 | 0.0 |  | 0.0 | 0.0 |
| West Bank |  | 0.0 | 0.0 |  | 4.4 | 6.6 |  | 0.0 | 0.0 |  | 1.1 | 0.0 |  | 2.6 | 0.0 |  | 0.0 | 0.0 |
| Madagascar |  | 0.0 | 38.2 |  | 9.0 | 0.1 |  | 0.0 | 0.0 |  | 0.0 | 0.0 |  | 31.0 | 0.0 |  | 0.0 | 0.0 |
| Sri Lanka |  | 0.1 | 3.9 |  | 0.0 | 0.0 |  | 0.0 | 0.0 |  | 0.0 | 0.0 |  | 5.7 | 0.0 |  | 0.0 | 0.0 |
| Timor-Leste |  | 0.5 | 1.8 |  | 0.0 | 0.1 |  | 0.0 | 0.0 |  | 0.0 | 0.0 |  | 3.4 | 0.0 |  | 0.0 | 0.0 |
| Rwanda |  | 5.2 | 9.2 |  | 0.7 | 0.2 |  | 0.0 | 0.0 |  | 0.1 | 0.2 |  | 6.1 | 0.0 |  | 0.1 | 0.7 |
| Lebanon |  | 0.2 | 0.7 |  | 8.8 | 10.3 |  | 0.0 | 0.0 |  | 0.5 | 0.5 |  | 4.0 | 0.0 |  | 0.0 | 0.0 |
| Israel |  | 0.0 | 0.2 |  | 12.8 | 17.7 |  | 0.0 | 0.0 |  | 2.4 | 0.0 |  | 6.2 | 0.2 |  | 0.0 | 0.0 |
| Central African Republic |  | 0.0 | 4.8 |  | 6.6 | 7.4 |  | 0.0 | 0.0 |  | 0.0 | 0.3 |  | 92.7 | 82.9 |  | 0.0 | 0.0 |
| Angola |  | 0.1 | 2.0 |  | 1.0 | 24.1 |  | 0.0 | 0.0 |  | 2.8 | 3.3 |  | 31.3 | 0.0 |  | 0.3 | 1.3 |
| Guinea-Bissau |  | 0.0 | 0.0 |  | 1.1 | 5.3 |  | 0.0 | 0.0 |  | 0.0 | 0.0 |  | 12.9 | 0.5 |  | 6.0 | 9.5 |
| Japan |  | 69.6 | 38.0 |  | 0.0 | 0.0 |  | 0.0 | 40.2 |  | 0.0 | 0.0 |  | 14.3 | 0.0 |  | 0.0 | 0.0 |
| Gabon |  | 2.4 | 12.8 |  | 0.0 | 0.0 |  | 0.0 | 0.0 |  | 0.0 | 0.0 |  | 30.8 | 2.8 |  | 2.2 | 14.0 |
| South Korea |  | 41.8 | 46.8 |  | 0.1 | 0.0 |  | 0.3 | 0.1 |  | 0.0 | 0.0 |  | 11.4 | 0.0 |  | 0.0 | 0.0 |
| Niger |  | 0.3 | 12.1 |  | 6.8 | 15.1 |  | 0.0 | 0.0 |  | 0.0 | 0.0 |  | 87.4 | 58.0 |  | 0.2 | 2.6 |
| Morocco |  | 0.0 | 40.1 |  | 0.0 | 15.1 |  | 0.2 | 0.4 |  | 23.2 | 0.0 |  | 39.3 | 0.0 |  | 0.0 | 0.0 |
| Mali |  | 0.0 | 10.5 |  | 32.3 | 35.0 |  | 0.0 | 0.0 |  | 0.0 | 0.0 |  | 94.9 | 71.7 |  | 1.1 | 1.8 |
| Tanzania |  | 0.3 | 73.2 |  | 26.4 | 1.4 |  | 0.0 | 0.0 |  | 14.6 | 11.5 |  | 57.4 | 0.3 |  | 0.0 | 0.1 |
| Burkina Faso |  | 0.2 | 146.0 |  | 34.5 | 0.3 |  | 0.0 | 0.0 |  | 0.0 | 0.0 |  | 127.4 | 0.0 |  | 0.2 | 2.5 |
| Congo, DRC |  | 7.1 | 79.5 |  | 8.0 | 2.2 |  | 0.0 | 0.0 |  | 0.3 | 3.2 |  | 306.6 | 71.6 |  | 79.5 | 229.0 |
| Malawi |  | 0.2 | 8.3 |  | 8.0 | 13.3 |  | 0.0 | 0.0 |  | 2.0 | 13.2 |  | 42.2 | 0.4 |  | 0.0 | 0.0 |
| Nicaragua |  | 7.2 | 26.1 |  | 0.9 | 0.0 |  | 0.0 | 0.0 |  | 0.0 | 0.0 |  | 49.7 | 1.0 |  | 2.5 | 3.9 |
| Chad |  | 0.0 | 14.7 |  | 16.9 | 26.1 |  | 0.0 | 0.0 |  | 0.0 | 0.9 |  | 133.5 | 77.1 |  | 0.0 | 0.0 |
| Ghana |  | 0.1 | 9.9 |  | 6.8 | 17.2 |  | 0.0 | 0.0 |  | 0.0 | 0.0 |  | 145.6 | 42.6 |  | 100.8 | 146.2 |
| Guinea |  | 0.1 | 2.2 |  | 9.5 | 107.2 |  | 0.0 | 0.0 |  | 0.0 | 0.0 |  | 122.2 | 0.7 |  | 70.2 | 3.2 |
| Senegal |  | 0.0 | 0.0 |  | 3.5 | 52.6 |  | 0.0 | 0.0 |  | 0.0 | 0.0 |  | 362.6 | 62.3 |  | 6.5 | 167.2 |
| Vietnam |  | 60.5 | 139.0 |  | 5.5 | 11.6 |  | 0.7 | 0.3 |  | 0.0 | 1.8 |  | 213.0 | 30.5 |  | 0.0 | 0.0 |
| Myanmar |  | 41.1 | 365.3 |  | 38.3 | 13.5 |  | 1.8 | 7.2 |  | 89.7 | 79.4 |  | 477.8 | 14.7 |  | 0.4 | 8.6 |
| Sudan |  | 0.0 | 29.9 |  | 45.4 | 358.1 |  | 0.0 | 0.1 |  | 2.7 | 29.0 |  | 770.4 | 0.6 |  | 0.0 | 0.0 |
| Nigeria |  | 134.4 | 1347.3 |  | 66.3 | 40.5 |  | 0.0 | 0.0 |  | 0.0 | 0.0 |  | 1696.7 | 329.9 |  | 678.0 | 22.6 |
| China |  | 5374.8 | 13555.2 |  | 2584.1 | 2989.2 |  | 3144.5 | 1602.7 |  | 557.0 | 827.6 |  | 9051.6 | 107.4 |  | 0.0 | 0.0 |
